# Risk for toxicity in older adults undergoing chemotherapy and its associated factors^1^

**DOI:** 10.1590/1518-8345.7910.4988

**Published:** 2026-11-13

**Authors:** Rianne Rodrigues de Lira, Alexandre de Souza Santos, Bruna Andrade Vigo, Jack Roberto Silva Fhon

**Affiliations:** 1 Universidade de São Paulo, Escola de Enfermagem, São Paulo, SP, Brazil.

**Keywords:** Aged, Geriatrics, Drug Therapy, Drug-Related Side Effects and Adverse Reactions, Toxicity, Nursing.

## Abstract

**Objective:**

to analyze the risk for toxicity and its associated factors in older adults undergoing chemotherapy.

**Method:**

a cross-sectional, analytical study comprising 179 participants. Data collection utilized a demographic profile form and a review of medical records, supplemented by validated instruments for geriatric and oncological assessment: 10-point Cognitive Screener, Geriatric Depression Scale, Hurria Toxicity Score, ECOG, Simple Questionnaire to Rapidly Diagnose Sarcopenia, Tilburg Frailty Indicator and the Social Support Scale. Data processing was based on inferential statistical methods, with a significance level of p < 0.05 adopted for all tests.

**Results:**

the study showed a predominance of women (58.4%), a mean age of 67.71, retirees (62.9%) and those with a caregiver (88.8%), cognitive impairment (31.4%), ECOG grades 2 and 3 (20.1%), risk for sarcopenia (33.7%), frailty (56.2%), depressive symptoms (23%), and moderate to high risk for toxicity (73.6%). It was found that older age, greater frailty, and poorer functional performance increase the risk for toxicity.

**Conclusion:**

the risk for toxicity associated with chemotherapy is present in older adults and is exacerbated by age, impaired functional performance, and frailty. This finding supports the need for better identification of nursing diagnoses, which can improve the care plan.

## Highlights

**(1)** Moderate and high toxicity risk was associated with older age.

**(2)** Moderate and high toxicity risk was associated with greater frailty.

**(3)** Moderate and high toxicity risk was associated with a decline in performance.

**(4)** Older adults with cancer and aged 60 to 70 have been understudied.

## Introduction

Demographic shifts have altered the population pyramid and led to an increased number of people aged 60 and older^1^. In 2020, the global cancer landscape recorded 19.3 million new cases, 64% of which affected individuals aged 60 or older - a group that accounted for 71.3% of cancer-related deaths. The most commonly diagnosed cancers were lung, colorectal, prostate, breast, and gastric cancers. Epidemiological projections indicate substantial worsening of this situation: it is estimated that, by 2040, the annual incidence will reach 20.7 million new cases, with 12.7 million deaths, highlighting the growing impact of neoplastic diseases on elderly populations worldwide^2^.

In the Brazilian epidemiological context, projections for the 2023-2025 period estimated an incidence of 483,000 new cancer cases. Analysis of the distribution by primary site revealed a predominance of breast (10.5%) and prostate (10.2%) cancers, followed, in terms of prevalence, by colon and rectal tumors (6.5%)^3^. These data underscore the need for public policies focused on the screening and early diagnosis of these diseases, which represent the primary burden of cancer-related morbidity in Brazil.

Oncological diseases, as well as their respective treatment protocols, have far-reaching consequences that go beyond the clinical setting, affecting the psychosocial and socioeconomic well-being of patients, families, and communities. It is observed that health systems in emerging economies (low- and middle-income countries) exhibit structural vulnerabilities in managing this epidemiological burden. This scenario is exacerbated by inequities in access, whereby a substantial portion of the global population remains marginalized regarding early diagnosis and timely, qualified therapeutic interventions, thus compromising prognosis and the sustainability of health services^4^.

Therapeutic modalities applied to adults and the elderly include isolated or combined approaches, based on surgical interventions, radiation therapy and specialized pharmacotherapy - the latter encompassing cytotoxic chemotherapy, hormone therapy, immunotherapy and molecularly targeted therapies. Within the scope of systemic treatments, antineoplastic chemotherapy is employed under different clinical guidelines as a curative modality; as an adjuvant, with the aim of eradicating residual micrometastases after definitive intervention; neoadjuvant, aiming for tumor downstaging prior to the main procedure; or, alternatively, as palliative care, for symptom control and prolongation of quality-adjusted survival^5^.

During the course of treatment, the patient is subject to the occurrence of adverse events, defined as deleterious and unintentional iatrogenic responses resulting from the administration of drugs at standard dosages for prophylactic, diagnostic or therapeutic purposes. Such complications require rigorous monitoring, since the systemic toxicity of antineoplastic agents often extends beyond the tumor target, affecting healthy tissues and requiring supportive interventions to maintain the viability of treatment^6^.

Such complications are inherent to the nonspecific mechanism of action of chemotherapy, which does not exhibit absolute selectivity for neoplastic cells. Toxicity manifests predominantly in tissues characterized by a high rate of cell proliferation, although the pharmacodynamics of these agents can trigger dysfunction in various organ systems. The magnitude of these reactions varies, depending on the specificity of the chemotherapy protocol, the patient’s functional reserve and the degree of systemic toxicity exerted on healthy cells^7^.

Measuring the toxicities resulting from cancer therapy is a fundamental step in the clinical practice of the healthcare team. For this purpose, the Common Terminology Criteria for Adverse Events (CTCAE) is adopted, a protocol established and periodically updated by the National Cancer Institute (NCI). This tool provides a descriptive taxonomy for classifying adverse events, stratifying them into five levels of severity based on clinical severity and functional impact, thereby enabling the standardization of management approaches and the comparability of data in clinical research^8^.

In 2018, the American Society of Clinical Oncology (ASCO) affirmed the indispensability of the Comprehensive Geriatric Assessment (CGA) for elderly patients undergoing chemotherapy. The guideline recommends that the application of this multidimensional tool is essential to inform personalized treatment planning, going beyond isolated chronological analysis. The implementation of CGA allows for risk stratification and the prediction of potentially preventable adverse events, optimizing patient safety and aiding clinical decision-making in the face of vulnerabilities intrinsic to aging^9^.

Studies have shown that the prevalence of toxicity in older adults is high, at 46%^10^ and 56.0%^11^, and the risk is associated with cognitive impairment^12^, decreased Body Mass Index (BMI)^10^ and the frailty syndrome^13^, which can pose health risks.

Chemotherapy-related toxicity in older adults is of critical importance due to its specific physiological characteristics, such as changes in renal, hepatic, and immune function, as well as potential comorbidities and the common occurrence of polypharmacy. These factors can increase the risk of adverse effects and complications during treatment, making it essential for nurses to understand these factors in order to provide safer and more effective care. By investigating such toxicity, nursing professionals can develop strategies for monitoring, early intervention, and patient education, thereby promoting more personalized care, minimizing side effects and improving the quality of life of older adults during cancer treatment. In this context, the objective of this study was to analyze the risk for toxicity and its associated factors in older adults undergoing chemotherapy.

## Method

### Study design 

A quantitative, observational, analytical, cross-sectional study.

### Study site 

The study was conducted at the São Paulo State Cancer Institute (ICESP), a unit within the *Hospital das Clínicas* complex of the University of São Paulo School of Medicine (HCFMUSP). The choice for the study setting is justified by the fact that it is a leading oncology center in Latin America, characterized by highly complex care and specialized services for the elderly population under the Unified Health System (SUS).

The hospital is located in the central region of the capital city, operates under a comprehensive care model through SUS and is responsible for meeting approximately 10% of the state’s oncology demand.

The outpatient antineoplastic therapy unit, located on the eleventh floor, has an infrastructure equipped with 107 infusion stations. This installed capacity enables the implementation of various therapeutic protocols, including the administration of chemotherapy, hormone therapy, immunotherapy and other systemic interventions^14^.

### Data collection period 

Data collection took place between July and October 2024, in the mornings and afternoons, Monday through Friday, and was conducted by the researcher and two assistants. All participants had previously been trained, through both virtual and in-person meetings, on how to administer and complete the questionnaires, and they were accompanied and supervised by the lead researcher at the start of data collection, during the initial approach to participants, and throughout the interviews.

### Population and sample 

The study population consisted of elderly patients in the initial phase of a chemotherapy regimen, including both treatment-naïve individuals and those starting a new treatment cycle following recurrence or disease progression. All participants were on the first day of the new treatment cycle. Convenience sampling was used, limited to the data collection period.

The selection process consisted of systematic screening conducted by the lead researcher using the chemotherapy department’s clinical schedule, where criteria such as age, current cycle, and treatment regimen were reviewed. Eligible patients were entered into a shared database, optimizing the planning of in-person visits and ensuring the systematization of the sample flow, in accordance with the institution’s care schedule.

During the data collection phase, 325 potential participants were screened. Of this total, 238 individuals were effectively recruited for the initial approach, of whom 22 declined to participate. Additionally, 17 individuals were excluded for not meeting the eligibility criteria and 20 because they did not undergo the therapeutic protocol on the scheduled date. Consequently, the final sample consisted of 179 participants.

### Selection criteria 

The following eligibility criteria were established for the study sample: a) individuals of either sex aged 60 years or older; b) histopathological diagnosis of primary solid neoplasm; c) initiation of intravenous chemotherapy, including both patients receiving first-line treatment and those undergoing a change in treatment regimen (line change) identified in the electronic medical record as C1D1, i.e., the first day of the first cycle of chemotherapy; and the ability to communicate and respond to questionnaires. The exclusion criteria were: having more than one concurrent oncological diagnosis under treatment; having an oncohematological diagnosis; and being enrolled in clinical research protocols.

### Study variables 

The study’s dependent variable, risk for toxicity, was analyzed as a numerical variable and was associated with demographic and clinical variables, cognitive status, functional performance, depressive symptoms, frailty, sarcopenia and social support.

### Data collection

Interviews were conducted with the participants after they had settled into the chair where their chemotherapy treatment would take place. The interviews occurred while the medication was being prepared for administration, over a period of approximately one hour. The interviews lasted an average of 35 to 45 minutes, which provided sufficient time to gather detailed and reliable information.

### Information collection instruments 

The following instruments were used:

In order to characterize the sample, a structured questionnaire was used to collect sociodemographic and economic data. This instrument allowed for the collection of key variables, including gender, chronological age, marital status, self-reported race or ethnicity and educational level. Additionally, indicators of living conditions and social support were investigated, such as current occupational activity, monthly family income, housing conditions, and the presence of support through a caregiver, ensuring a comprehensive understanding of the social determinants that may influence the clinical outcome of older adults.

Clinical data were collected through the analysis of medical records, aiming to identify key oncological variables for the analysis of prognosis and therapeutic risk. Information regarding the primary site of the neoplasm, initial clinical staging, current line of treatment and the prescribed chemotherapy protocol was extracted. Additionally, the history of prior exposure to immunotherapy and the eligibility criteria for the initiation of cytotoxic chemotherapy were examined, thereby ensuring an accurate record of each participant’s therapeutic trajectory.

Cognitive performance was assessed using the 10-point Cognitive Screener (10-CS), developed in Brazil and used as a rapid measure of cognitive performance. The test consists of subtests of temporal orientation (day, month, and year) (0 to 3 points), learning and recall of three objects (0 to 3 points) and categorical verbal fluency (animals in 60 seconds) (0 to 4 points).

Two points are added for individuals with no schooling, and one point is added for individuals with one to three years of schooling. The total score ranges from zero to 10, classifying the participant as: normal (10-8 points), possible cognitive impairment (6-7 points), and probable cognitive impairment (0-5 points)^15^.

Depressive symptoms were assessed using the Geriatric Depression Scale (GDS), which has been validated for Brazilian Portuguese^16^. The scale consists of 15 dichotomous items (Yes and No); the score ranges from 0 to 15, with a cutoff point of ≥ 5 indicating the likely presence of depressive symptoms.

The risk for toxicity was identified using Hurria’s Toxicity Score, which has been translated and validated in Brazil^17^. This is a predictive tool for assessing toxicity risk, encompassing 11 variables, namely: age 72 years or older, cancer of the gastrointestinal or genitourinary tract, number of chemotherapy agents, hemoglobin less than 11 g/dL for men and less than 10 g/dL for women, creatinine clearance less than 34 mL/min, borderline hearing or severe hearing loss, one or more falls in the past six months, taking medications with partial assistance or being unable to do so, walking one block with moderate or severe limitation and a decrease in social activities, at least on some occasions, due to physical and/or emotional limitations^17^.

Creatinine clearance was calculated using the Cockcroft-Gault formula, employing the electronic calculator provided by the Brazilian Society of Nephrology (SBN). For the calculation, body weight, chronological age and the serum creatinine value obtained at the time of approval of the chemotherapy protocol were considered. Similarly, hemoglobin levels were extracted from laboratory tests associated with the authorization of cytotoxic therapy. Toxicity risk stratification was established based on the sum of the scores of the predictive variables, allowing participants to be classified into three categories: low risk (0-5 points), moderate risk (6-9 points) and high risk (10-19 points)^17^.

Functional status was assessed using the Eastern Cooperative Oncology Group Performance Status (ECOG-PS) scale. This instrument, widely validated in clinical oncology, measures the patient’s degree of autonomy on a five-point scale: grade 0 (fully active); grade 1 (restricted from strenuous activities, but capable of performing light work); grade 2 (capable of self-care, but unable to perform work activities, remaining out of bed for more than 50% of waking hours); grade 3 (limited self-care, remaining in bed or a chair for more than 50% of waking hours); and grade 4 (total inability to perform self-care and complete confinement to bed)^18^.

Sarcopenia was identified using the Simple Questionnaire to Rapidly Diagnose Sarcopenia (SARC-F), which has been validated for Brazil and consists of five objective questions regarding strength, walking, the ability to stand up from a chair, climbing a flight of stairs and a history of falls and, in addition, by measuring calf circumference, yielding a score ranging from 0 to 20 points. As a result, participants can be classified as having no signs suggestive of sarcopenia (0 to 10 points) or signs suggestive of sarcopenia (11 to 20 points)^19^.

Frailty assessment was conducted using the Tilburg Frailty Indicator (TFI), a validated instrument that has been cross-culturally adapted into Portuguese. TFI is a multidimensional scale comprising 15 objective and self-reported items, structured to investigate three fundamental domains: physical, psychological, and social. The total score is obtained by summing the responses, ranging from 0 to 15 points. A cutoff point of five points or less was adopted for classifying the frailty phenotype, as recommended by the literature, to identify individuals with greater clinical vulnerability^20^.

Perception of social support was measured using the Medical Outcomes Study Social Support Survey (MOS-SSS), in its version adapted and validated for the Brazilian population. The instrument consists of 19 items that assess the frequency of support availability through the central question: “If you need it, how often can you count on someone?” Responses are structured on a five-point Likert scale, ranging from 0 (“never”) to 4 (“always”). Given the absence of specific normative values for the Brazilian context, the analysis was based on the premise of proportionality, in which higher total scores indicate a greater perception of social support in the domains investigated^21^.

### Data analysis 

Data processing and statistical analysis were performed using the Statistical Package for the Social Sciences (SPSS) software, version 25.0. The normality of the numerical variables was tested using the Kolmogorov-Smirnov test. To characterize the sample, a descriptive analysis was performed, in which categorical variables were expressed as absolute and relative frequencies. In turn, continuous variables were summarized using measures of central tendency and dispersion, specifically mean and standard deviation.

Subsequently, bivariate analyses were conducted to investigate associations with the outcome “risk for toxicity”, treated as the dependent variable. For cross-tabulations between categorical variables, Pearson’s chi-square test was applied. To compare means across different groups, Student’s t-test for independent samples was used to identify statistical significance in the correlations established between predictor factors and vulnerability to the adverse event.

To determine the magnitude of the associations between the risk for toxicity (dependent variable) and the predictor factors, a multiple linear regression model was applied. The independent variables included demographic profile (age, gender, marital status, presence of a caregiver, education level, parity, income and social support), health indicators (comorbidities), and clinical domains (functional capacity, cognitive status, depressive symptoms, sarcopenia and frailty). In all inferential analyses, a significance level of p < 0.05 and a 95% confidence interval (CI) were adopted.

### Ethical aspects

The research protocol was submitted to and approved by the Scientific Committee for Teaching and Research at ICESP (registration NP5049/23) and by the Research Ethics Committee of *Hospital das Clínicas* of the University of São Paulo School of Medicine (CEP-HCFMUSP), under the Certificate of Submission for Ethical Review (CAAE) No. 78467124.2.0000.0068.

The study was conducted in strict compliance with the ethical principles established by Resolution No. 466/2012 of the National Health Council, which governs research involving human subjects. Data collection began only after all participants had given their consent and signed the Informed Consent Form (ICF), ensuring anonymity and voluntary participation.

## Results

The study population was predominantly female (58.4%), aged 60-69 (63.5%) with a mean age of 67.7 (± 5.63) years, married (47.2%) and retired (62.9%); with an average monthly income of R$ 2,709.84 (= 2,426.50) and an average of 8.66 (= 4.54) years of schooling, home owners (71.9%) and those with a caregiver (88.8%) (Table 1).

**Table 1:** Sociodemographic characteristics of older adults on the first day of chemotherapy (n = 179). São Paulo, SP, Brazil, 2024

Variable	Category	n* (%)	Mean (= SD^†^)
Sex	Female	104 (58.4)	
	Male	74 (41.6)	
Age	60 - 69	113 (63.5)	67.71 (= 5.63)
	70 - 79	58 (32.6)	
	80 or over	7 (3.9)	
Marital status	Married	84 (47.2)	
	Single	34 (19.1)	
	Widowed	33 (18.5)	
	Divorced	21 (11.8)	
	Separated	5 (2.8)	
	Commom-law relationship	1 (0.6)	
Self-reported color or race	White	96 (53.9)	
	*Pardo* (multi-racial)	46 (25.8)	
	Black	32 (18.0)	
	Indigenous	2 (1.1)	
	Asian	2 (1.1)	
Current occupation	Retired	112 (62.9)	
	Unemployed	35 (19.7)	
	Self-employed	19 (10.7)	
	Employed	12 (6.7)	
Education			8.66 (= 4.54)
Montly income			2,709.84^‡^ (= 2,426.55)
Presence of a caregiver	Yes	158 (88.8)	
	No	20 (11.2)	
Housing type	Own house	128 (71.9)	
	Rented house	27 (15.2)	
	Living in the main caregiver’s house	20 (11.2)	
	Other	3 (1.7)	

*n = Number; ^†^SD = Standard Deviation; ^‡^Current minimum wages = R$ 1,412.00, Brazil, 2024

The average number of comorbidities was found to be 1.26 (SD = 1.09) conditions, with the most prevalent being systemic hypertension (50.6%), diabetes mellitus (28.1%), dyslipidemia (21.3%) and heart disease and hypothyroidism (both at 3.4%).

The most common cancer diagnosis among participants, according to medical records, was breast cancer (18.5%), followed by bronchial and lung cancer (12.9%), colon cancer (11.8%), rectal cancer (7.3%), pancreatic cancer (6.2%), stomach (3.9%), oropharynx and esophagus (3.4% each).

Regarding the characterization of treatment modalities, the majority of the sample (82.2%) had no history of immunotherapy exposure, while 11.8% had received this treatment. Regarding chemotherapy regimens, first-line chemotherapy was predominant (33.7%), followed by adjuvant (18.5%) and neoadjuvant (11.3%) regimens. Subsequent treatment lines were identified in smaller proportions: second-line (9.6%), third-line (6.7%), and fourth-line (2.2%). Additionally, therapeutic intentions and specific protocols were recorded as palliative treatment (9.0%), induction therapy (5.1%), standard protocols (3.9%) and curative intent (1.7%)

With regard to clinical assessment, it was found that participants had possible cognitive impairment (20.8%) and probable cognitive impairment (9.6%), with ECOG category 2 (19.1%), at risk for sarcopenia (33.7%), frail (56.2%), moderate (48.9%) and high (24.7%) risk for toxicity, and depressive symptoms (23.0%). Regarding social support, high perception was found in the domain of material support (84.3%), affective support (89.3%), emotional support (75.8%) and positive social interaction (69.7%) (Table 2).

**Table 2:** Characterization of the assessment of older adults on the first day of chemotherapy (n = 179). São Paulo, SP, Brazil, 2024

Variable	Category	Mean (=SD*)	n^†^	%
Cognitive status	Normal	7.89 (1.86)	124	69.7
	Possible cognitive impairment		37	20.8
	Probable cognitive impairment		17	9.6
ECOG-PS^‡^	0	0.93 (0.72)	51	28.7
	1		91	51.1
	2		34	19.1
	3		2	1.1
Sarcopenia	No risk for sarcopenia	6.92 (5.69)	118	66.3
	At risk for sarcopenia		60	33.7
Frailty	Not frail	5.11 (2.36)	79	43.8
	Frail		100	56.2
Hurria	Low	7.57 (3.03)	47	26.4
	Moderate		87	48.9
	High		44	24.7
Depressive symptoms	No symptoms	2.96 (2.59)	137	77.0
	With symptoms		41	23.0
MOSS-SSS^§^	Material support	14.67 (3.00)		
	High perception		150	84.3
	Medium perception		22	12.4
	Low perception		6	3.4
	Affective support	11.39 (1.93)		
	High perception		159	89.3
	Medium perception		14	7.9
	Low perception		5	2.8
	Emotional support	26.88 (9.71)		
	High perception		135	75.8
	Medium perception		20	11.2
	Low perception		23	12.9
	Positive social interaction	12.65 (5.37)		
	High perception		124	69.7
	Medium perception		21	11.8
	Low perception		33	18.5

*SD = Standard Deviation; ^†^n = Number; ^‡^ECOG-PS = Eastern Cooperative Oncology Group Performance Status Scale; ^§^MOSS-SSS = Social Support Scale

In the analysis of the relationship between toxicity outcomes and categorical independent variables, Student’s t-test showed a statistically significant difference in the risk for developing adverse chemotherapy events based on gender (p = 0.04) (Table 3).

**Table 3:** Association between the risk for chemotherapy-related toxicity and gender, marital status and the presence of a caregiver among older adults on the first day of chemotherapy treatment (n = 179). São Paulo, SP, Brazil, 2024

Variable	Category	Mean	=SD*	p-value^†^
Sex	Female	7.16	2.78	0.04
	Male	8.14	3.03	
Marital status	With a partner	7.99	3.14	0.07
	Without a partner	7.18	2.89	
Has a caregiver	Yes	7.71	2.95	0.14
	No	6.45	3.53	

*SD = Standard Deviation; ^†^p-value = Statistical significance

As for the correlation analysis, it was found that the risk for toxicity correlated with age (p < 0.001), ECOG (p < 0.001), depressive symptoms (p < 0.001), sarcopenia (p < 0.001), and frailty (p < 0.001) (Table 4).

Association analyses showed that the risk for chemotherapy toxicity was significantly influenced by biological and functional variables. It was observed that advancing age (p = 0.001), increased frailty scores (p = 0.012) and a decline in performance status, as measured by the RCOG-PS (p = 0.003), were positive predictors of increased vulnerability to adverse events during treatment (Table 5).

**Table 4:** Correlation between the risk for chemotherapy-related toxicity and sociodemographic and clinical variables among older adults on the first day of chemotherapy treatment (n = 179). São Paulo, SP, Brazil, 2024

Variable	r*	p-value†
Age	0.339	<0.001
Education level	-0.092	0.220
Number of children	0.081	0.283
Income of the older adult	-0.039	0.602
Total comorbidities	0.078	0.301
ECOG-PS^‡^	0.429	<0.001
Total cognitive status	-0.119	0.113
Total depressive symptoms	0.291	<0.001
Total sarcopenia	0.332	<0.001
Total frailty	0.336	<0.001
MOSS-SSS^§^ Material support	0.045	0.549
MOSS-SSS^§^ Affective support	-0.143	0.058
MOSS-SSS^§^ Emotional support	-0.088	0.243
MOSS-SSS^§^ Positive social interaction	-0.126	0.093

*r = Pearson’s correlation; **†**p-value = Statistical significance; ^‡^ECOG-PS = Eastern Cooperative Oncology Group Performance Status Scale; ^§^MOSS-SSS = Social Support Scale

**Table 5:** Association between the risk for chemotherapy-related toxicity and sociodemographic and clinical variables among older adults on the first day of chemotherapy treatment (n = 179). São Paulo, SP, Brazil, 2024

Variable	B*	t^†^	p-value^‡^	CI 95%^§^ low^||^ and upp^¶^	
Age	0.128	3.379	0.001	0.053	0.202
Sex	-0.622	-1.434	0.154	-1.479	0.235
Marital status	-0.486	-1.171	0.244	-1.305	0.334
Has a caregiver	1.054	1.549	0.123	0.290	2.397
Education level	0.043	0.846	0.399	-0.057	0.143
Number of children	0.028	0.235	0.815	-0.208	0.265
Income	-5.793	-0.655	0.513	0.000	0.000
Total comorbidities	0.048	0.243	0.808	-0.340	0.436
ECOG-PS**	0.957	2.983	0.003	0.323	1.590
Cognitive status	0.065	0.548	0.585	-0.171	0.301
Depressive symptoms	0.124	1.410	0.161	-0.050	0.297
Total sarcopenia	0.064	1.644	0.102	-0.013	0.141
Frailty	0.238	2.550	0.012	0.054	0.422
MOS-SSS^††^ - Material support	0.077	0.999	0.319	-0.075	0.229
MOS-SSS^††^ - Affective support	-0.055	-0.485	0.628	-0.279	0.169
MOS-SSS^††^ - Emotional support	-0.025	-1.080	0.282	-0.070	0.020
MOS-SSS^††^ - Positive social interaction	-0.040	-0.988	0.325	-0.121	0.040

*B = Beta; ^†^t = t-value; ^‡^p-value = Statistical significance; ^§^95% CI = Confidence interval; ^||^Lower; ^¶^Upper; **ECOG-PS = Eastern Cooperative Oncology Group Performance Status Scale; ^††^MOSS-SSS = Social Support Scale

## Discussion

This study highlights important information regarding older adults undergoing chemotherapy, which can contribute to better management and care planning for these patients. The study found that older adults who are frail and have reduced functional performance are at greater risk for toxicity when undergoing chemotherapy.

Regarding the study population, it consisted mainly of women, with a mean age of 67.7 years, a predominance of the 60-69 age group, and married individuals. Similar studies have identified divergent results regarding gender^22^ ^-^ ^23^. As for age, the means were alike^22^ ^-^ ^23^_,_ which is also true for married individuals^21^ ^,^ ^24^.

As for the prevalence of toxicity risk, nearly three-quarters of the sample presented moderate to high risk. A Chinese study found that, among 253 elderly cancer patients, 24.17% exhibited toxicity^25^. On the other hand, a systematic review identified a prevalence of toxicity of 45.7% in the study population for all non-hematological cancers^26^.

When interpreting the clinical significance of these data, it should be considered that, in most cases, non-hematological toxicity has an impact on the patient’s quality of life, interfering with functional capacity, emotional well-being, cognitive function and the use of healthcare resources and hospitalization, leading to an increased risk for institutionalization^27^.

Studies have shown that the use of specific tools in the assessment of older adults, such as CGA, reduces the risk for chemotherapy toxicity and improves quality of life^28^.

In the specific context of chemotherapy, CGA serves as a clinical stratification tool, allowing the elderly patient’s profile to be categorized into three distinct phenotypes: (a) robust patients, whose functional reserve permits the administration of conventional protocols similar to those used for younger age groups; (b) frail patients, in whom the accumulation of comorbidities and functional decline suggest that the benefits of cytotoxic chemotherapy are outweighed by the risks, prioritizing palliative approaches; and (c) vulnerable patients, who exhibit impairments in specific CGA domains, requiring personalized gerontological interventions and prophylactic measures to optimize their therapeutic viability^27^. It should be noted that the systematic application of this tool can be effectively carried out by nursing professionals, thereby strengthening advanced practice and risk management in oncology. 

The association analyses in this study confirm chronological age as a determining factor, showing that advancing age is directly proportional to vulnerability to adverse events. However, the literature presents controversies. A retrospective cohort study conducted in Korea observed divergent data when stratifying the sample into age groups (under and over 70 years), failing to identify a significant association between age alone and the risk for toxicity^23^. This disparity may be attributed to the biological heterogeneity of aging, since older adults present heightened risks of functional impairment and multimorbidity. Such conditions, more than chronological age *per se*, result in reduced physiological reserve and greater interference with activities of daily living, increasing the complexity of therapeutic management^29^.

Oncological management in senescence and old age remains a multifactor therapeutic challenge, conditioned by the marked clinical heterogeneity of this age group. This complexity stems from variability in levels of independence, burden of comorbidities and functional reserve - elements that hinder the standardization of therapeutic decisions. Additionally, physiological aging imposes significant pharmacokinetic and pharmacodynamic changes, reducing tissue selectivity and narrowing the therapeutic index of drugs, which increases the risk for severe toxicity. The prevalence of multimorbidity and polypharmacy exacerbate such vulnerability, thereby increasing the likelihood of harmful drug interactions. Finally, the scarcity of robust clinical trials specifically focused on the elderly population results in an evidence gap regarding the efficacy and safety of antineoplastic protocols, limiting the basis for the optimal management of toxicities^30^.

Most of the study population was categorized as frail and associated with a risk for toxicity. A study found that 43.0% of participants were classified as frail^24^. On the other hand, another study found that older adults considered frail were not associated with a risk for toxicity^22^. Frailty is conceptualized as a multidimensional clinical syndrome, characterized by a marked reduction in physiological reserve and the capacity to resist stressors. This condition results from a cumulative decline in physiological systems, manifesting as a decrease in muscle strength and organ function. Consequently, a state of increased vulnerability is observed, which predisposes the individual to adverse outcomes, such as a decline in autonomy (dependence), an increased burden of morbidity and increased mortality rates^31^.

Identifying pre-frail and frail patients is essential for facilitating more targeted and effective supportive care interventions and assessments. This practice has important clinical implications, as, although it is possible to recognize frail patients, changes in pre-frail patients tend to be more subtle and difficult to detect. As a result, there is a risk of missing opportunities for early personalized guidance and for conducting more rigorous monitoring during treatment, especially in patients who appear more robust than they actually are^32^.

The dynamics of the frailty syndrome are guided by transitional mechanisms that must be carefully inferred from clinical observation. Evidence suggests that tumor progression, the toxicity inherent in anticancer treatment and the exacerbation of pre-existing comorbidities or acute complications act as triggers for the development or worsening of such condition. Although frailty is a dynamic condition and amenable to intervention, the guidelines of the International Society of Geriatric Oncology (SIOG) and the American Society of Clinical Oncology (ASCO) emphasize the need for continuous monitoring during and after the treatment cycle^33^ ^-^ ^34^. In this context, the technical training of nurses becomes a strategic cornerstone, enabling the early identification of functional decline and the implementation of specialized monitoring guidelines, which are essential for risk mitigation and the promotion of safety for elderly patients.

In the context of routine clinical practice, longitudinal monitoring of frailty - based on periodic reassessments following the initiation of the oncology regimen and integrated with non-oncological geriatric interventions - constitutes an indispensable tool for measuring the patient’s responsiveness to the treatment plan. This continuous monitoring provides critical insights for the multidisciplinary team to dynamically adjust care strategies, allowing for the maintenance, intensification, de-escalation or discontinuation of interventions according to the progression of the clinical condition. This approach enables the strategic redirection of treatment focus toward emerging vulnerabilities, which may supersede the priorities identified in the pre-treatment stage, ensuring personalized care centered on each elderly person’s clinical trajectory^35^.

With regard to functional status, the sample in this study focused on ECOG grades 2 and 3, demonstrating that impaired performance status acts as a direct predictor of increased toxicity risk in the elderly population. In contrast, although a German study, conducted with 44 older adults, identified a prevalence of 15.9% for the same ECOG grades (2 and 3), it did not observe a statistically significant association with the risk for adverse events (p = 0.310)^22^. Furthermore, another study identified a positive correlation between the frailty syndrome, assessed by the frailty index, and ECOG in elderly cancer patients, with a 0.10-point increase on the frailty scale when the patient is classified as ECOG 0 to 1^24^.

Exploratory safety analysis in clinical trials based on the ECOG scale supports the premise that functional decline is a predictor of unfavorable outcomes. It was observed that increasing rates of adverse events are intrinsically associated with impaired performance status, resulting in high rates of premature treatment discontinuation. This scenario of functional vulnerability not only limits adherence to the anticancer protocol, but also acts as a determining factor for clinical deterioration or death, reinforcing the need for rigorous monitoring in patients with reduced functional reserve. In a statement, ASCO recommended including patients with reduced functional capacity in clinical trials, as this limits available treatment options^36^.

As the cancer progresses, functional capacity tends to decline sharply, severely affecting quality of life and establishing itself as a robust independent predictor of survival. This impairment is exacerbated by systemic inflammation and ineffective symptom management. Biomarkers, such as elevated C-reactive protein (CRP) and cancer cachexia, act as critical mediators in the deterioration of functional reserve in older adults^37^. Although ECOG-PS is widely adopted in clinical oncology due to its practical applicability and clinical efficiency, the literature warns that this tool may be insufficient to capture the nuances of geriatric and gerontological performance, neglecting specific domains that constitute the multidimensionality of aging^30^.

The study has limitations such as the method, which, being cross-sectional, does not allow for causality identification regarding the risk for toxicity. Furthermore, statistical inference is not possible because the sample was selected for convenience. Despite these limitations, the results provide important insights into older adults and how nurses can intervene before toxicity occurs.

These findings are extremely important for nursing because they highlight factors that can aid in the early identification of the most vulnerable patients to chemotherapy-induced toxicity. By recognizing that older adults who are frailer and have poorer functional status are at higher risk for developing adverse effects, the nursing team can monitor these patients more closely and tailor care and interventions to their specific needs. Such actions contribute to safer, more individualized and more efficient care for older adults undergoing chemotherapy, in addition to enabling early interventions that can minimize potential toxicities, improve the patient’s quality of life, and consequently optimize the outcomes of cancer treatment.

## Conclusion

The study results showed that the risk for toxicity was moderate to high in the sample, and that it was associated with the participant’s older age, greater frailty and a worse ECOG score.

These results highlight the need to improve the process of identifying potential risks for chemotherapy-related toxicity in elderly patients on the first day of chemotherapy, contributing to a better care plan and, consequently, enhanced care for this population during treatment. The identification of such risks can be conducted in the nursing team’s clinical practice, as the team has the first point of contact and already performs pre-treatment assessments, such as venous access evaluation, in order to mitigate the risk of extravasation, for example. Screening for this type of risk can be performed using the Hurria instrument and comprehensive geriatric assessment, administered during the nursing consultation prior to the start of the first chemotherapy cycle, with the purpose of identifying vulnerabilities in elderly patients.

Given the results, it is essential to highlight the importance of a differentiated and attentive approach to this population in order to promote better treatment outcomes, reduce toxicities and treatment interruptions, and thereby improve the quality of life of older adults during the therapeutic period. Furthermore, the need for studies with larger samples is emphasized, as these can provide a deeper understanding and confirm the findings of the present study. It is also important to invest in research using varied methodologies, aiming to develop interventions based on solid and clear evidence that is appropriate to the specificities of the elderly population, with the goal of improving care and offering safer and more effective clinical practice.

## Data Availability

Datasets related to this article will be available upon request to the corresponding author.

## References

[1] Ministério do Desenvolvimento e Assistência Social, Família e Combate à Fome (BR) (2023). Nota Informativa nº 5/2023: envelhecimento e o direito ao cuidado [Internet].

[2] Li L, Shan T, Zhang D, Ma F (2024). Nowcasting and forecasting global aging and cancer burden: analysis of data from the GLOBOCAN and Global Burden of Disease Study. J Natl Cancer Cent.

[3] Santos MO, Lima FCS, Martins LFL, Oliveira JFP, Almeida LM, Cancela MC (2023). Estimated cancer incidence in Brazil, 2023-2025. Rev Bras Cancerol.

[4] World Health Organization (2020). Decade of healthy ageing: plan of action [Internet]..

[5] Anand U, Dey A, Chandel AKS, Sanyal R, Mishra A, Pandey DK (2023). Cancer chemotherapy and beyond: current status, drug candidates, associated risks and progress in targeted therapeutics. Genes Dis.

[6] United Nations (2002). Political declaration and Madrid international plan of action on ageing [Internet].

[7] Liu B, Zhou H, Tan L, Siu KTH, Guan X (2024). Exploring treatment options in cancer: tumor treatment strategies. Signal Transduct Target Ther.

[8] National Cancer Institute (2017). Common terminology criteria for adverse events (CTCAE) [Internet]..

[9] Mohile SG, Dale W, Somerfield MR, Schonberg MA, Boyd CM, Burhenn PS (2018). Practical assessment and management of vulnerabilities in older patients receiving chemotherapy: ASCO guideline for geriatric oncology. J Clin Oncol.

[10] Hamid M, Hannan M, Oo NM, Lynch P, Walsh DJ, Matthews T (2022). Chemotherapy toxicity in older adults optimized by geriatric assessment and intervention: a non-comparative analysis. Curr Oncol.

[11] Dhir V, Maganti M, Rozenberg D, Kukreti V, Kuruvilla J, Crump M (2022). Predicting the risks of aggressive-intent chemotherapy toxicity in older patients with lymphoma: a prospective observational pilot study. Clin Lymphoma Myeloma Leuk.

[12] Min GJ, Cho BS, Park SS, Park S, Jeon YW, Shin SH (2022). Geriatric assessment predicts nonfatal toxicities and survival for intensively treated older adults with AML. Blood.

[13] Baltussen JC, de Glas NA, van Holstein Y, van der Elst M, Trompet S, Uit den Boogaard A (2023). Chemotherapy-related toxic effects and quality of life and physical functioning in older patients. JAMA Netw Open.

[14] Instituto do Câncer do Estado de São Paulo (2025). Especialidades [Internet].

[15] Apolinario D, Lichtenthaler DG, Magaldi RM, Soares AT, Busse AL, Amaral JR (2016). Using temporal orientation, category fluency, and word recall for detecting cognitive impairment: the 10-point cognitive screener (10-CS). Int J Geriatr Psychiatry.

[16] Almeida OP, Almeida SA (1999). Reliability of the Brazilian version of the Geriatric Depression Scale (GDS) short. Arq Neuropsiquiatr.

[17] Pontes LB, Chinaglia L, Karnaskis T, Todaro J, Rodrigues HV, Souza PMR (2017). Chemotherapy in the elderly: translation of Hurria’s toxicity score to Portuguese. Geriatr Gerontol Aging.

[18] Oken MM, Creech RH, Tormey DC, Horton J, Davis TE, McFadden ET (1982). Toxicity and response criteria of the Eastern Cooperative Oncology Group. Am J Clin Oncol.

[19] Barbosa-Silva TG, Menezes AM, Bielemann RM, Malmstrom TK, Gonzalez MC (2016). Enhancing SARC-F: improving sarcopenia screening in the clinical practice. J Am Med Dir Assoc.

[20] Santiago LM, Luz LL, Mattos IE, Gobbens RJJ (2012). Cross-cultural adaptation of the Tilburg Frailty Indicator (TFI) for use in the Brazilian population. Cad Saude Publica.

[21] Zanini DS, Verolla-Moura AV, Queiroz IPAR (2009). Social support: validity aspects of the construct in under graduation student. Psicol Estudo.

[22] Akbıyık I, Yüce D, Kars A, Halil MG (2022). Association of frailty with chemotherapy toxicity in chemotherapy-naive older patients with cancer. Support Care Cancer.

[23] Jeong HT, Kim HG, Han J (2024). Efficacy and toxicity of palliative chemotherapy in elderly patients with advanced pancreatic cancer. Pancreas.

[24] Fletcher J, Reid N, Hubbard RE, Berry R, Weston M, Walpole E (2024). Frailty index, not age, predicts treatment outcomes and adverse events for older adults with cancer. J Frailty Aging.

[25] Hua Y, Zou Y, Guan M, Yuan HY, Zhou Y, Liu F (2023). Predictive model of chemotherapy-related toxicity in elderly Chinese cancer patients. Front Pharmacol.

[26] Han CJ, Ning X, Burd CE, Spakowicz DJ, Tounkara F, Kalady MF (2024). Chemotoxicity and associated risk factors in colorectal cancer: a systematic review and meta-analysis. Cancers.

[27] Feliu J, Heredia-Soto V, Gironés R, Jiménez-Munarriz B, Saldaña J, Guillén-Ponce C (2020). Management of the toxicity of chemotherapy and targeted therapies in elderly cancer patients. Clin Transl Oncol.

[28] Disalvo D, Moth E, Soo WK, Garcia MV, Blinman P, Steer C (2023). The effect of comprehensive geriatric assessment on care received, treatment completion, toxicity, cancer-related and geriatric assessment outcomes, and quality of life for older adults receiving systemic anti-cancer treatment: a systematic review. J Geriatr Oncol.

[29] Flannery MA, Culakova E, Canin BE, Peppone L, Ramsdale E, Mohile SG (2021). Understanding treatment tolerability in older adults with cancer. J Clin Oncol.

[30] Badic B, Oguer M, Cariou M, Kermarrec T, Bouzeloc S, Nousbaum JB (2021). Prognostic factors for stage III colon cancer in patients 80 years of age and older. Int J Colorectal Dis.

[31] Morley JE, Vellas B, van Kan GA, Anker SD, Bauer JM, Bernabei R (2013). Frailty consensus: a call to action. J Am Med Dir Assoc.

[32] Lee HJ, Boscardin J, Walter LC, Smith AK, Cohen HJ, Giri S (2025). Associations of frailty with survival, hospitalization, functional decline, and toxicity among older adults with advanced non-small cell lung cancer. Oncologist.

[33] Loh KP, Liposits G, Arora SP, Neuendorff NR, Gomes F, Krok-Schoen JL (2024). Adequate assessment yields appropriate care-the role of geriatric assessment and management in older adults with cancer: a position paper from the ESMO/SIOG Cancer in the Elderly Working Group. ESMO Open.

[34] Diaz FC, Hamparsumian A, Loh KP, Verduzco-Aguirre H, Abdallah M, Williams GR (2024). Geriatric oncology: a 5-year strategic plan. Am Soc Clin Oncol Educ Book.

[35] Goede V. (2023). Frailty and cancer: current perspectives on assessment and monitoring. Clin Interv Aging.

[36] Abi Jaoude J, Kouzy R, Mainwaring W, Lin TA, Miller AB, Jethanandani A (2020). Performance status restriction in phase III cancer clinical trials. J Natl Compr Canc Netw.

[37] Muhandiramge J, Orchard SG, Warner ET, van Londen GJ, Zalcberg JR (2022). Functional decline in the cancer patient: a review. Cancers.

