# Supplementary material for: Risk for toxicity in older adults undergoing chemotherapy and its associated factors
Source: Rev Lat Am Enfermagem. 2026 Nov 13;34:e4988. doi: 10.1590/1518-8345.7910.4988 (PMC13762696; doi:10.1590/1518-8345.7910.4988)
Supplement: Translation

# Translation: Risco para toxicidade na pessoa idosa em tratamento quimioterápico e seus fatores associados^*^

**DOI:** 10.1590/1518-8345.7910.4989

**Authors:** Rianne Rodrigues de Lira, Alexandre de Souza Santos, Bruna Andrade Vigo, Jack Roberto Silva Fhon

**Affiliations:** 1 Universidade de São Paulo, Escola de Enfermagem, São Paulo, SP, Brasil.

**Keywords:** Idoso, Geriatria, Tratamento Farmacológico, Efeitos Colaterais e Reações Adversas Relacionados a Medicamentos, Toxicidade, Enfermagem.

## Abstract

**Objetivo:**

analisar o risco de toxicidade e seus fatores associados na pessoa idosa em tratamento quimioterápico.

**Método:**

estudo de delineamento transversal e analítico, composto por 179 participantes. A coleta de dados utilizou formulário de perfil demográfico e consulta a prontuários, complementada por instrumentos validados para a avaliação geriátrica e oncológica: *10-point Cognitive Screener*, Escala de Depressão Geriátrica, Escore de Toxicidade de Hurria, o ECOG, *Simple Questionnaire to Rapidly Diagnose Sarcopenia*, Índice de Fragilidade de Tilburg e a Escala de Apoio Social. O processamento dos dados fundamentou-se em métodos de estatística analítica inferencial, adotando-se, para todos os testes, um nível de significância de p < 0,05.

**Resultados:**

o estudo apresentou predomínio de mulheres (58,4%), idade média de 67,71, aposentados (62,9%) e com cuidador (88,8%), comprometimento cognitivo (31,4%), ECOG grau 2 e 3 (20,1%), risco de sarcopenia (33,7%), fragilidade (56,2%), sintomas depressivos (23%) e moderado e alto risco de toxicidade (73,6%). Identificou-se que maior idade, maior fragilidade e pior performance funcional aumentam o risco de toxicidade.

**Conclusão:**

o risco de toxicidade associado à quimioterapia está presente na pessoa idosa; acrescenta-se à idade piora na performance e fragilidade. Tal verificação fortalece a melhor identificação de diagnósticos de enfermagem, podendo melhorar o plano de cuidados.

## Destaques

**(1)** Risco de toxicidade moderado e alto foi associado a maior idade.

**(2)** Risco de toxicidade moderado e alto foi associado a maior fragilidade.

**(3)** Risco de toxicidade moderado e alto foi associado a piora de performance.

**(4)** Pessoas idosas oncológicas com idade de 60 a 70 anos são pouco estudadas.

## Introdução

Com as mudanças demográficas, tem-se modificado a pirâmide populacional e aumentou o número de pessoas acima de 60 anos^1^. Em 2020, o cenário oncológico global registrou 19,3 milhões de casos incidentes, dos quais 64% acometeram indivíduos na faixa etária igual ou superior a 60 anos - grupo que concentrou 71,3% da mortalidade associada a essas patologias. As neoplasias de maior prevalência diagnóstica abrangeram os sítios pulmonar, colorretal, prostático, mamário e gástrico. Projeções epidemiológicas indicam um agravamento substancial dessa conjuntura: estima-se que, até 2040, a incidência anual alcance 20,7 milhões de novos casos, com um contingente de 12,7 milhões de óbitos, evidenciando o impacto crescente das doenças neoplásicas nas populações idosas em escala mundial^2^. 

No contexto epidemiológico brasileiro, as projeções para o triênio 2023-2025 estimaram uma incidência de 483 mil novos casos oncológicos. A análise da distribuição por sítios primários revelou uma predominância das neoplasias de mama (10,5%) e próstata (10,2%), seguidas, em termos de prevalência, pelos tumores de cólon e reto (6,5%)^3^. Tais dados reiteram a necessidade de políticas públicas direcionadas ao rastreamento e diagnóstico precoce dessas patologias, que representam as principais cargas de morbidade neoplásica no território nacional.

As patologias oncológicas, bem como seus respectivos protocolos terapêuticos, deflagram repercussões multidimensionais que transcendem o quadro clínico, incidindo sobre as esferas psicossocial e socioeconômica de pacientes, núcleos familiares e coletividades. Observa-se que os sistemas de saúde em nações de economias emergentes (baixa e média renda) apresentam vulnerabilidades estruturais na gestão desse ônus epidemiológico. Tal cenário é agravado pela iniquidade no acesso, em que uma parcela substancial da população global permanece marginalizada quanto ao diagnóstico precoce e a intervenções terapêuticas qualificadas em janelas de tempo oportunas, comprometendo o prognóstico e a sustentabilidade dos serviços de saúde^4^.

As modalidades terapêuticas aplicadas a pessoas adultas e idosas compreendem abordagens isoladas ou combinadas, fundamentadas em intervenções cirúrgicas, radioterapia e farmacoterapia especializada - esta última abrangendo a quimioterapia citotóxica, hormonioterapia, imunoterapia e terapias de alvo molecular. No âmbito dos tratamentos sistêmicos, a quimioterapia antineoplásica é empregada sob diferentes diretrizes clínicas como modalidade curativa; adjuvante, com o intuito de erradicar micrometástases residuais após a intervenção definitiva; neoadjuvante, visando ao *downstaging* tumoral prévio ao procedimento principal; ou, ainda, em caráter paliativo, para o controle de sintomas e extensão da sobrevida com qualidade^5^.

No decurso do tratamento, o paciente está sujeito à manifestação de eventos adversos, conceituados como respostas iatrogênicas deletérias e não intencionais, decorrentes da administração de fármacos em posologias habituais para fins profiláticos, diagnósticos ou terapêuticos. Tais intercorrências exigem monitoramento rigoroso, uma vez que a toxicidade sistêmica dos agentes antineoplásicos frequentemente transcende o alvo tumoral, impactando tecidos hígidos e demandando intervenções de suporte para a manutenção da viabilidade do tratamento^6^.

Tais intercorrências são inerentes ao mecanismo de ação inespecífico da quimioterapia, a qual não possui seletividade absoluta pelas células neoplásicas. A toxicidade manifesta-se predominantemente em tecidos caracterizados por alta taxa de proliferação celular embora a farmacodinâmica desses agentes possa desencadear disfunções em diversos sistemas orgânicos. A magnitude dessas reações é variável, dependendo da especificidade do protocolo quimioterápico, da reserva funcional do paciente e pelo grau de toxicidade sistemática exercida sobre as células saudáveis^7^.

A mensuração das toxicidades decorrentes da terapêutica oncológica constitui uma etapa fundamental na prática clínica da equipe assistencial. Para tal finalidade, adota-se o *Common Terminology Criteria for Adverse Events* (CTCAE), protocolo estabelecido e periodicamente atualizado pelo *National Cancer Institute* (NCI). Essa ferramenta provê uma taxonomia descritiva para a classificação de eventos adversos, estratificando-os em cinco níveis de gravidade de acordo com a severidade clínica e o impacto funcional, permitindo a padronização das condutas de manejo e a comparabilidade de dados em pesquisas clínicas^8^.

Em 2018, a *American Society of Clinical Oncology* (ASCO) ratificou a imprescindibilidade da Avaliação Geriátrica Ampla (AGA) para pacientes idosos sob regime quimioterápico. A diretriz preconiza que a aplicação dessa ferramenta multidimensional é essencial para fundamentar o planejamento terapêutico personalizado, transcendendo a análise cronológica isolada. A implementação da AGA permite a estratificação de riscos e a predição de eventos adversos potencialmente evitáveis, otimizando a segurança do paciente e auxiliando na tomada de decisão clínica frente às vulnerabilidades intrínsecas ao envelhecimento^9^. 

Estudos tem demonstrado que a prevalência de toxicidade na pessoa idosa é alta, 46%^10^ e 56,0%^11^ e o risco está associado ao comprometimento cognitivo^12^, diminuição do Índice de Massa Corpórea (IMC)^10^ e síndrome da fragilidade^13^ o que pode trazer riscos à saúde.

A toxicidade devido à quimioterapia na pessoa idosa é extremamente importante devido às suas especificidades fisiológicas, como alterações na função renal, hepática e imunológica, além de possíveis comorbidades e a presença comum de polifarmácia. Essas particularidades podem aumentar o risco de efeitos adversos e complicações durante o tratamento, tornando essencial que a enfermagem compreenda esses fatores para oferecer cuidados mais seguros e eficazes. Ao investigar essa toxicidade, a enfermagem pode desenvolver estratégias de monitoramento, intervenção precoce e educação do paciente, promovendo uma assistência mais personalizada, minimizando efeitos colaterais e melhorando a qualidade de vida da pessoa idosa durante o tratamento oncológico. Nesse sentido, o objetivo de estudo foi analisar o risco de toxicidade e seus fatores associados na pessoa idosa em tratamento quimioterápico. 

## Método

### Tipo do estudo 

Estudo quantitativo, observacional, analítico e de corte transversal. 

### Local de estudo 

O estudo foi conduzido no Instituto do Câncer do Estado de São Paulo (ICESP), unidade integrante do complexo do Hospital das Clínicas da Faculdade de Medicina da Universidade de São Paulo (HCFMUSP). A escolha do cenário de pesquisa justifica-se por tratar-se de um centro de referência em oncologia na América Latina, caracterizado pela alta complexidade assistencial e pelo atendimento especializado à população idosa sob os regimes do Sistema Único de Saúde (SUS).

A instituição hospitalar está situada na região central da capital, opera sob regime de atendimento integral pelo Sistema Único de Saúde (SUS), sendo responsável pela assistência de aproximadamente 10% da demanda oncológica do estado.

A unidade de terapia antineoplásica ambulatorial, alocada no décimo primeiro andar, dispõe de uma infraestrutura dimensionada com 107 postos de infusão. Essa capacidade instalada permite a viabilização de diversos protocolos terapêuticos, englobando a administração de quimioterapia, hormonioterapia, imunoterapia e demais intervenções sistêmicas^14^.

### Período de coleta de dados 

A coleta foi realizada entre julho e outubro de 2024, nos períodos da manhã e tarde, de segunda a sexta-feira, pelo pesquisador e dois colaboradores, todos foram previamente capacitados, por meio de reuniões virtuais e presenciais, para aplicação e preenchimento dos instrumentos, e acompanhados e supervisionados no início da coleta, nos momentos de abordagem e seguimento das entrevistas pelo pesquisador principal. 

### População e amostra 

A população do estudo foi composta por pacientes idosos em fase inicial de protocolo quimioterápico, incluindo tanto indivíduos novos no tratamento (*naïve*) quanto aqueles em início de um novo ciclo terapêutico após recidiva ou progressão, estando todos os indivíduos no primeiro dia do ciclo do novo tratamento. Adotou-se uma amostragem por conveniência, delimitada pelo período de coleta de dados.

O processo de seleção consistiu em uma triagem sistemática conduzida pela pesquisadora principal a partir da agenda clínica do setor de quimioterapia, onde eram verificados critérios como idade, ciclo atual e regime terapêutico. Os pacientes elegíveis eram inseridos em uma base de dados compartilhada, otimizando o planejamento das abordagens presenciais e garantindo a sistematização do fluxo amostral, conforme o cronograma assistencial da instituição.

Durante a etapa de coleta de dados, procedeu-se ao rastreio de 325 participantes potenciais. Desse montante, 238 indivíduos foram efetivamente recrutados para abordagem inicial, dos quais 22 manifestaram recusa à participação. Adicionalmente, 17 indivíduos foram excluídos por não atenderem aos critérios de elegibilidade e 20 devido à não realização do protocolo terapêutico na data prevista. Consequentemente, a amostra definitiva foi constituída por 179 participantes.

### Critérios de seleção 

Para a composição da amostra do estudo, estabeleceram-se os seguintes critérios de elegibilidade: a) indivíduos de ambos os sexos com idade cronológica igual ou superior a 60 anos; b) diagnóstico histopatológico de neoplasia sólida primária; c) início de protocolo quimioterápico por via intravenosa, contemplando tanto pacientes em primeira linha de tratamento quanto aqueles submetidos à alternância de regime terapêutico (troca de linha) identificado em prontuário eletrônico como C1D1, ou seja, primeiro dia do primeiro ciclo de tratamento quimioterápico; e capacidade de comunicar e responder aos questionários. Já os critérios de exclusão foram: ter mais de um diagnóstico oncológico em tratamento concomitante; ter diagnóstico oncohematológico; e estar inserido em protocolos de pesquisa clínica.

### Variáveis do estudo 

A variável dependente do estudo, risco de toxicidade, foi analisada como numérica e foi associada a variáveis demográficas, clínicas, estado cognitivo, performance funcional, sintomas depressivos, fragilidade, sarcopenia e apoio social.

### Coleta de dados

Foram realizadas entrevistas com os participantes após sua acomodação na poltrona onde seria realizado o tratamento quimioterápico. A abordagem ocorreu enquanto o medicamento estava sendo preparado para administração, em um período de aproximadamente uma hora. As entrevistas tiveram uma duração média entre 35 e 45 minutos, o que garantiu um momento adequado para coleta de informações detalhadas e confiáveis.

### Instrumentos para a coleta das informações

Foram utilizados os seguintes instrumentos:

Para a caracterização da amostra, foi utilizado um formulário estruturado destinado ao levantamento do perfil sociodemográfico e econômico. Esse instrumento permitiu a coleta de variáveis fundamentais, incluindo sexo, idade cronológica, estado civil, cor ou raça autodeclarada e nível de escolaridade. Adicionalmente, foram investigados indicadores do contexto de vida e suporte social, tais como a atividade ocupacional atual, renda mensal familiar, configuração da moradia e a presença de suporte por meio de cuidador, garantindo uma compreensão abrangente das determinantes sociais que podem influenciar o desfecho clínico da pessoa idosa. 

A coleta de dados clínicos foi realizada mediante análise de prontuários, visando ao levantamento de variáveis oncológicas fundamentais para a análise do prognóstico e do risco terapêutico. Foram extraídas informações referentes ao sítio primário da neoplasia, estadiamento clínico inicial, linha de tratamento atual e o protocolo quimioterápico prescrito. Adicionalmente, verificou-se o histórico de exposição prévia à imunoterapia e os critérios de elegibilidade para a liberação da quimioterapia citotóxica, assegurando o registro preciso da trajetória terapêutica de cada participante.

O desempenho cognitivo foi avaliado utilizando o *10-point Cognitive Screener* (10-CS), criado no Brasil e utilizado como medida rápida de desempenho cognitivo. O teste é composto por subtestes de orientação temporal (dia, mês e ano) (0 a 3 pontos), aprendizado e evocação de três objetos (0 a 3 pontos) e fluência verbal categórica (animais em 60 segundos) (0 a 4 pontos).

Dois pontos são adicionados para indivíduos não escolarizados e um ponto é adicionado para indivíduos com um a três anos de escolaridade. O escore total varia de zero a 10 classificando ao participante em: normal (10 - 8 pontos), comprometimento cognitivo possível (6 - 7 pontos) e comprometimento cognitivo provável (0 - 5 pontos)^15^.

Procedeu-se à avaliação de sintomas depressivos utilizando a Escala de Depressão Geriátrica (GDS), que foi validada para o português do Brasil^16^. A escala consta de 15 questões dicotômicas (Sim e Não); o escore varia de 0 a 15 e apresenta o ponto de corte ≥ 5 como indicativo dos casos de provável presença de sintomas depressivos.

A identificação do risco de toxicidade foi realizada por meio do Escore de toxicidade de Hurria, traduzido e validado no Brasil^17^. Trata-se de um instrumento preditivo para a avaliação de risco de toxicidade, englobando 11 variáveis, sendo elas: idade maior ou igual a 72 anos, câncer do trato gastrointestinal ou geniturinário, número de quimioterápicos, hemoglobina menor que 11 g/dL para homens e menor que 10 g/dL para mulheres, *clearance* de creatinina menor que 34 mL/min, audição limítrofe ou déficit auditivo grave, uma ou mais quedas nos últimos seis meses, tomar medicações com ajuda parcial ou não conseguir, caminhar um quarteirão com limitação moderada ou acentuada, diminuição das atividades sociais, pelo menos em algumas ocasiões, devido à limitação física e/ou emocional^17^.

O cálculo do *clearance* de creatinina foi realizado por meio da fórmula de Cockcroft-Gault, utilizando-se a calculadora eletrônica disponibilizada pela Sociedade Brasileira de Nefrologia (SBN). Para o processamento do cálculo, foram considerados o peso corporal, a idade cronológica e o valor da creatinina sérica obtido no momento da liberação do protocolo quimioterápico. Da mesma forma, os níveis de hemoglobina foram extraídos dos exames laboratoriais vinculados à autorização da terapia citotóxica. A estratificação do risco de toxicidade foi estabelecida a partir do somatório da pontuação das variáveis preditoras, permitindo a classificação dos participantes em três categorias: baixo risco (0 - 5 pontos), risco moderado (6 - 9 pontos) e alto risco (10 - 19 pontos)^17^.

A avaliação do *status* funcional foi realizada por meio da escala *Eastern Cooperative Oncology Group Performance Status* (ECOG-PS). Esse instrumento, amplamente validado na oncologia clínica, mensura o grau de autonomia do paciente em cinco níveis: grau 0 (completamente ativo); grau 1 (restrição a atividades rigorosas, mas capaz de realizar trabalhos leves); grau 2 (capaz de realizar o autocuidado, mas impossibilitado de atividades laborais, permanecendo fora do leito por mais de 50% do tempo acordado); grau 3 (autocuidado limitado, permanecendo no leito ou cadeira por mais de 50% do período de vigília); e grau 4 (incapacidade total de autocuidado e confinamento integral ao leito)^18^.

A identificação da sarcopenia fez-se com uso do *Simple Questionnaire to Rapidly Diagnose Sarcopenia* (SARC-F), validado para o Brasil e conformado por cinco perguntas objetivas sobre força, deambulação, ato de levantar-se de uma cadeira, subir um lance de escadas e ter sofrido quedas e, além disso, com a verificação da circunferência da panturrilha, podendo possuir valor de 0 a 20 pontos. Como resultado, pode-se classificar o participante em sem sinais sugestivos de sarcopenia (0 a 10 pontos) e sugestivo de sarcopenia (11 a 20 pontos)^19^.

A avaliação da fragilidade foi conduzida por meio do Índice de Fragilidade de Tilburg (TFI), instrumento validado e adaptado transculturalmente para a língua portuguesa. O TFI caracteriza-se como uma escala multidimensional composta por 15 itens objetivos e autorreferidos, estruturados para investigar três domínios fundamentais: físico, psicológico e social. A pontuação global é obtida pelo somatório das respostas, variando em um intervalo de 0 a 15 pontos. Adotou-se o ponto de corte de menor ou igual a cinco pontos para a classificação do fenótipo de fragilidade, conforme preconizado pela literatura, para a identificação de indivíduos com maior vulnerabilidade clínica^20^. 

A percepção do suporte social foi mensurada pela *Medical Outcomes Study Social Support Survey* (MOS-SSS), em sua versão adaptada e validada para a população brasileira. O instrumento é constituído por 19 itens que avaliam a frequência de disponibilidade de apoio por meio da indagação central: “Se você precisar, com que frequência conta com alguém?”. As respostas são estruturadas em uma escala do tipo Likert de cinco pontos, variando de 0 (“nunca”) a 4 (“sempre”). Dada a ausência de valores normativos específicos para o contexto brasileiro, a análise fundamentou-se na premissa de proporcionalidade, na qual escores totais mais elevados indicam uma percepção superior de suporte social nos domínios investigados^21^.

### Análise dos dados 

O processamento e a análise estatística dos dados foram viabilizados pelo *software Statistical Package for the Social Sciences* (SPSS), versão 25.0. A aderência das variáveis numéricas à distribuição normal foi verificada por meio do teste de Kolmogorov-Smirnov. Para a caracterização da amostra, procedeu-se à análise descritiva, na qual as variáveis categóricas foram expressas em frequências absolutas e relativas. Por sua vez, as variáveis contínuas foram sintetizadas por meio de medidas de tendência central e de dispersão, especificamente média e desvio-padrão.

Subsequentemente, foram conduzidas análises bivariadas para investigar as associações com o desfecho ‘risco de toxicidade’, tratado como variável dependente. No cruzamento entre variáveis categóricas, aplicou-se o teste de Qui-quadrado de Pearson. Para a comparação das médias entre grupos distintos, utilizou-se o teste t de Student para amostras independentes, visando a identificar significância estatística nas correlações estabelecidas entre os fatores preditores e a vulnerabilidade ao evento adverso.

Para determinar a magnitude das associações entre o risco de toxicidade (variável dependente) e os fatores preditores, aplicou-se o modelo de regressão linear múltipla. As variáveis independentes incluíram o perfil demográfico (idade, gênero, estado civil, presença de cuidador, escolaridade, paridade, renda e apoio social), indicadores de saúde (comorbidades) e domínios clínicos (capacidade funcional, estado cognitivo, sintomatologia depressiva, sarcopenia e fragilidade). Em todas as análises inferenciais, adotou-se o nível de significância p < 0,05 e intervalo de confiança (IC) de 95%.

### Aspectos éticos 

O protocolo de pesquisa foi submetido e aprovado pela Comissão Científica de Ensino e Pesquisa do ICESP (registro NP5049/23) e pelo Comitê de Ética em Pesquisa do Hospital das Clínicas da Faculdade de Medicina da Universidade de São Paulo (CEP-HCFMUSP), sob o Certificado de Apresentação de Apreciação Ética (CAAE) nº 78467124.2.0000.0068. 

O estudo foi conduzido em estrita observância aos preceitos éticos estabelecidos pela Resolução n.º 466/2012 do Conselho Nacional de Saúde, que rege as pesquisas envolvendo seres humanos. A coleta de dados foi iniciada somente após a anuência e assinatura do Termo de Consentimento Livre e Esclarecido (TCLE) por todos os participantes, assegurando o anonimato e a voluntariedade da colaboração.

## Resultados

Identificou-se na população estudada o predomínio do sexo feminino (58,4%), entre 60 e 69 (63,5%) com idade média de 67,7 (= 5,63) anos, casados (47,2%), aposentados (62,9%), a média da renda mensal era de R$ 2.709,84 (= 2.426,50) e da escolaridade de 8,66 (= 4,54) anos, com casa própria (71,9%) e com cuidador (88,8%) (Tabela 1).

**Tabela 1:** Características sociodemográficas das pessoas idosas em primeiro dia de tratamento quimioterápico (n = 179). São Paulo, SP, Brasil, 2024

Variável	Categoria	n* (%)	Média (=DP^†^)
Sexo	Feminino	104 (58,4)	
	Masculino	74 (41,6)	
Idade	60 - 69	113 (63,5)	67,71 (= 5,63)
	70 - 79	58 (32,6)	
	80 ou mais	7 (3,9)	
Estado civil	Casado	84 (47,2)	
	Solteiro	34 (19,1)	
	Viúvo	33 (18,5)	
	Divorciado	21 (11,8)	
	Separado	5 (2,8)	
	Relação estável	1 (0,6)	
Cor ou raça autodeclarada	Branco	96 (53,9)	
	Pardo	46 (25,8)	
	Preto	32 (18,0)	
	Indígena	2 (1,1)	
	Amarelo	2 (1,1)	
Atividade atual	Aposentado	112 (62.9)	
	Desempregado	35 (19.7)	
	Autônomo	19 (10,7)	
	Vínculo empregatício	12 (6,7)	
Escolaridade			8,66 (= 4,54)
Renda mensal			2.709,84^‡^ (= 2.426,55)
Presença de cuidador	Sim	158 (88,8)	
	Não	20 (11,2)	
Tipo de moradia	Casa própria	128 (71,9)	
	Alugada	27 (15,2)	
	Casa do cuidador principal	20 (11,2)	
	Outros	3 (1,7)	

*n = Número; ^†^DP = Desvio-Padrão; ^‡^Salário-mínimo vigente = R$ 1.412,00, Brasil, 2024

Identificou-se que a média de comorbidades foi de 1,26 (DP=1,09) doenças, sendo as mais prevalentes de hipertensão arterial sistêmica (50,6%), diabetes mellitus (28,1%), dislipidemia (21,3%) e cardiopatia e hipotireoidismo (ambos com 3,4%).

O diagnóstico oncológico mais presente nos participantes, de acordo com o registro da consulta médica foi o câncer de mama (18,5%), brônquios e dos pulmões (12,9%), cólon (11,8%), reto (7,3%), pâncreas (6,2%), estômago (3,9%), orofaringe e esófago (3,4$, respectivamente). 

Quanto à caracterização das modalidades terapêuticas, a maioria da amostra (82,2%) não apresentava histórico de exposição à imunoterapia, enquanto 11,8% haviam recebido tal modalidade. No que tange aos protocolos quimioterápicos, observou-se uma predominância da quimioterapia de primeira linha (33,7%), seguida pelos regimes de adjuvância (18,5%) e neoadjuvância (11,3%). As linhas subsequentes de tratamento foram identificadas em menores proporções: segunda linha (9,6%), terceira linha (6,7%) e quarta linha (2,2%). Adicionalmente, as intenções terapêuticas e protocolos específicos foram registrados como tratamento paliativo (9,0%), terapia de indução (5,1%), protocolos padrão (3,9%) e intenção curativa (1,7%)

No que diz respeito à avaliação clínica, foi evidenciado que os participantes tinham comprometimento cognitivo possível (20,8%) e comprometimento cognitivo provável (9,6%), com ECOG categoria 2 (19.1%), com risco de sarcopenia (33,7%), frágeis (56,2%), risco de toxicidade moderado (48,9%) e alto (24,7%) com sintomas depressivos (23,0%). Relativo ao apoio social, foi encontrado no domínio apoio material com alta percepção (84,3%), apoio afetivo com alta percepção (89,3%), apoio emocional com alta percepção com 75,8%, e interação social positiva com alta percepção (69,7%) (Tabela 2).

**Tabela 2:** Caracterização da avaliação das pessoas idosas em primeiro dia de tratamento quimioterápico (n = 179). São Paulo, SP, Brasil, 2024

Variável	Categoria	Média (=DP*)	n^†^	%
Estado cognitivo	Normal	7,89 (1,86)	124	69,7
	Comprometimento cognitivo possível		37	20,8
	Comprometimento cognitivo provável		17	9,6
ECOG-PS^‡^	0	0,93 (0,72)	51	28.7
	1		91	51.1
	2		34	19.1
	3		2	1.1
Sarcopenia	Sem risco de sarcopenia	6,92 (5,69)	118	66,3
	Com risco de sarcopenia		60	33,7
Fragilidade	Não frágil	5,11 (2,36)	79	43,8
	Frágil		100	56,2
Hurria	Baixo	7,57 (3,03)	47	26,4
	Moderado		87	48,9
	Alto		44	24,7
Sintomas depressivos	Sem sintomas	2,96 (2,59)	137	77,0
	Com sintomas		41	23,0
MOSS-SSS^§^	Apoio material	14,67 (3,00)		
	Alta percepção		150	84,3
	Média percepção		22	12,4
	Baixa percepção		6	3,4
	Apoio afetivo	11,39 (1,93)		
	Alta percepção		159	89,3
	Média percepção		14	7,9
	Baixa percepção		5	2,8
	Apoio emocional	26,88 (9,71)		
	Alta percepção		135	75,8
	Média percepção		20	11,2
	Baixa percepção		23	12,9
	Interação social positiva	12,65 (5,37)		
	Alta percepção		124	69,7
	Média percepção		21	11,8
	Baixa percepção		33	18,5

*DP = Desvio-Padrão; ^†^n = Número; ^‡^ECOG-PS = *Eastern Cooperative Oncologic Group Performance Status Scale*; ^§^MOSS-SSS = Escala de Apoio Social

Na investigação das relações entre o desfecho de toxicidade e as variáveis independentes categóricas, a aplicação do teste t de *Student* revelou uma disparidade estatisticamente significante no risco de desenvolvimento de eventos adversos quimioterápicos em função do sexo (p = 0,04) (Tabela 3).

**Tabela 3:** Relação entre risco de toxicidades relacionada à quimioterapia com o gênero, estado civil e presença de cuidador nas pessoas idosas em primeiro dia de tratamento quimioterápico (n = 179). São Paulo, SP, Brasil, 2024

Variável	Categoria	Média	=DP*	p-valor^†^
Sexo	Feminino	7,16	2,78	0,04
	Masculino	8,14	3,03	
Estado civil	Com companheiro	7,99	3,14	0,07
	Sem companheiro	7,18	2,89	
Tem cuidador	Sim	7,71	2,95	0,14
	Não	6,45	3,53	

*DP = Desvio-Padrão; ^†^p-valor = Significância estatística

Quanto ao teste de correlação, foi evidenciado que o risco de toxicidade foi correlacionado com a idade (p < 0,001), ECOG (p < 0,001), sintomas depressivos (p < 0,001), sarcopenia (p < 0,001) e fragilidade (p < 0,001) (Tabela 4).

As análises de associação revelaram que o risco de toxicidade quimioterápica foi significativamente influenciado por variáveis biológicas e funcionais. Observou-se que o avanço da idade (p = 0,001), o incremento nos escores de fragilidade (p = 0,012) e o declínio do *status* de desempenho, mensurado pelo ECOG (p = 0,003), constituíram-se como fatores preditores positivos para o aumento da vulnerabilidade a eventos adversos durante o tratamento. (Tabela 5).

**Tabela 4:** Correlação entre o risco de toxicidade relacionada à quimioterapia com variáveis sociodemográficas e clínicas das pessoas idosas em primeiro dia de tratamento quimioterápico (n = 179). São Paulo, SP, Brasil, 2024

Variável	r*	p-valor^†^
Idade	0,339	<0,001
Escolaridade	-0,092	0,220
Número de filhos	0,081	0,283
Renda da pessoa idosa	-0,039	0,602
Total de comorbidades	0,078	0,301
ECOG-PS^‡^	0,429	<0,001
Total do estado cognitivo	-0,119	0,113
Total sintomas depressivos	0,291	<0,001
Total sarcopenia	0,332	<0,001
Total fragilidade	0,336	<0,001
MOSS-SSS^§^ Apoio material	0,045	0,549
MOSS-SSS^§^ Apoio afetivo	-0,143	0,058
MOSS-SSS^§^ Apoio emocional	-0,088	0,243
MOSS-SSS^§^ Interação social positiva	-0,126	0,093

*r = Correlação de Pearson; ^†^p-valor = Significância estatística; ^‡^ECOG-PS = *Eastern Cooperative Oncologic Group Performance Status Scale*; ^§^MOSS-SSS= Escala de Apoio Social

**Tabela 5:** Associação entre o risco de toxicidade relacionada à quimioterapia e variáveis sociodemográficas e clínicas das pessoas idosas em primeiro dia de tratamento quimioterápico (n = 179). São Paulo, SP, Brasil, 2024

Variável	B*	t^†^	p-valor^‡^	IC 95%^§^ inf^||^ e sup^¶^	
Idade	0,128	3,379	0,001	0,053	0,202
Sexo	-0,622	-1,434	0,154	-1,479	0,235
Estado civil	-0,486	-1,171	0,244	-1,305	0,334
Tem cuidador	1,054	1,549	0,123	0,290	2,397
Escolaridade	0,043	0,846	0,399	-0,057	0,143
Número de filhos	0,028	0,235	0,815	-0,208	0,265
Renda	-5,793	-0,655	0,513	0,000	0,000
Total de comorbidades	0,048	0,243	0,808	-0,340	0,436
ECOG-PS**	0,957	2,983	0,003	0,323	1,590
Estado cognitivo	0,065	0,548	0,585	-0,171	0,301
Sintomas depressivos	0,124	1,410	0,161	-0,050	0,297
Total sarcopenia	0,064	1,644	0,102	-0,013	0,141
Fragilidade	0,238	2,550	0,012	0,054	0,422
MOS-SSS^††^ -Apoio material	0,077	0,999	0,319	-0,075	0,229
MOS-SSS^††^ - Apoio afetivo	-0,055	-0,485	0,628	-0,279	0,169
MOS-SSS^††^ - Apoio emocional	-0,025	-1,080	0,282	-0,070	0,020
MOS-SSS^††^ - Interação social positiva	-0,040	-0,988	0,325	-0,121	0,040

*B = Beta; ^†^t = Valor t; ^‡^p-valor = Significância estatística; ^§^IC95% = Índice de confiança; ^||^inf = Inferior; ^¶^sup = Superior; **ECOG-PS = *Eastern Cooperative Oncologic Group Performance Status Scale*; ^††^MOSS-SSS = Escala de apoio social

## Discussão

Este estudo realça informações importantes, relacionadas à população idosa em tratamento quimioterápico, podendo contribuir para um melhor manejo e planejamento do cuidado a esse paciente. Neste estudo identificou-se que a pessoa idosa com mais idade, que apresenta diminuição da performance da funcionalidade e é frágil, tem maior risco de toxicidade quando está sob tratamento quimioterápico.

Quanto à população do estudo, esta foi conformada majoritariamente por mulheres com a média da idade de 67,7 anos, com o predomínio da faixa etária entre 60 e 69 anos e dos casados. Estudos similares identificaram resultados divergentes em relação ao gênero^22^ ^-^ ^23^. Quanto à idade, as médias foram semelhantes^22^ ^-^ ^23^, e igualmente quanto aos casados^21^ ^,^ ^24^. 

Em relação à prevalência de risco de toxicidade, quase três quartas partes da amostra apresentavam um risco moderado e alto. Um estudo chinês identificou, em 253 pacientes idosos com câncer, que 24,17% apresentavam toxicidade^25^. Por outro lado, uma revisão sistemática identificou, para todos os tipos de câncer não hematológicas, a prevalência de toxicidade de 45,7% na população do estudo^26^. Ao interpretar o valor clínico desses dados, deve-se considerar que, na maioria dos casos, a toxicidade não hematológica tem um impacto na qualidade de vida do paciente, interferindo com a capacidade funcional, a esfera emocional, a função cognitiva e o uso de recursos de saúde e hospitalização, levando a um risco aumentado de institucionalização^27^.

Estudos têm identificado que o uso de ferramentas especificas na avaliação da pessoa idosa, como a AGA, reduz o risco de toxicidade por quimioterapia e melhora a qualidade de vida^28^. 

No domínio específico da quimioterapia, a AGA atua como um instrumento de estratificação clínica, que permite categorizar o perfil da pessoa idosa em três fenótipos distintos: (a) pacientes robustos, cuja reserva funcional permite a administração de protocolos convencionais análogos aos de faixas etárias mais jovens; (b) pacientes frágeis, nos quais o acúmulo de comorbidades e o declínio funcional sugerem que o benefício da quimioterapia citotóxica é sobreposto pelos riscos, priorizando-se abordagens paliativas; e (c) pacientes vulneráveis, que apresentam prejuízos em domínios específicos da AGA, demandando intervenções gerontológicas personalizadas e medidas profiláticas para otimizar sua viabilidade terapêutica^27^. Ressalta-se que a aplicação sistemática dessa ferramenta pode ser efetivamente conduzida pelo profissional de enfermagem, fortalecendo a prática avançada e o gerenciamento de riscos na oncologia.

As análises de associação do presente estudo ratificam a idade cronológica como um fator determinante, evidenciando que o avanço etário guarda relação diretamente proporcional com a vulnerabilidade a eventos adversos. Contudo, a literatura apresenta controvérsias; um estudo de coorte retrospectiva conduzido na Coreia observou dados divergentes ao estratificar a amostra em grupos etários (menores e maiores de 70 anos), não identificando associação significante entre a idade isolada e o risco de toxicidade^23^. Essa disparidade pode ser atribuída à heterogeneidade biológica do envelhecimento, uma vez que as pessoas idosas apresentam riscos acentuados de comprometimento funcional e multimorbidades. Tais condições, mais do que a idade cronológica *per se*, resultam em uma menor reserva fisiológica e maior interferência nas atividades de vida diária, elevando a complexidade do manejo terapêutico^29^.

O manejo oncológico na senescência e senilidade permanece como um desafio terapêutico multifatorial, condicionado pela marcante heterogeneidade clínica desse estrato etário. Essa complexidade decorre da variabilidade nos níveis de independência, carga de comorbidades e reserva funcional, elementos que dificultam a padronização das decisões terapêuticas. Adicionalmente, as senescências fisiológicas impõem alterações farmacocinéticas e farmacodinâmicas significativas, reduzindo a seletividade tecidual e estreitando o índice terapêutico dos fármacos, o que potencializa o risco de toxicidade grave. A prevalência de multimorbidades e o regime de polifarmácia exacerbam essa vulnerabilidade, elevando a probabilidade de interações medicamentosas deletérias. Por fim, a escassez de ensaios clínicos robustos focados especificamente na população idosa resulta em uma lacuna de evidências sobre a eficácia e a segurança dos protocolos antineoplásicos, limitando o embasamento para o manejo otimizado das toxicidades^30^.

A maior parte da população estudada foi categorizada como frágil e associado ao risco de toxicidade. Um estudo identificou que 43,0% dos participantes foram classificados como frágeis^24^. Por outro lado, outro estudo identificou que aquelas pessoas idosas consideradas frágeis não foram associadas com o risco de toxicidade^22^. A fragilidade é conceituada como uma síndrome clínica multidimensional, caracterizada pela redução acentuada da reserva fisiológica e da capacidade de resistência a estressores. Essa condição decorre de um declínio cumulativo nos sistemas fisiológicos, manifestando-se pela diminuição da força muscular e da funcionalidade orgânica. Como consequência, observa-se um estado de vulnerabilidade aumentada, que predispõe o indivíduo a desfechos adversos, tais como o declínio da autonomia (dependência), o agravamento da carga de morbidade e a elevação dos índices de mortalidade^31^. 

A identificação de pacientes pré-frágeis e frágeis é fundamental para facilitar intervenções e avaliações de cuidados de suporte mais direcionados e eficazes. Essa prática possui implicações clínicas importantes, pois, embora seja possível reconhecer pacientes frágeis, as mudanças em pacientes pré-frágeis tendem a ser mais sutis e difíceis de detectar. Como resultado, há o risco de perder oportunidades de orientação antecipada personalizada e de realizar um monitoramento mais rigoroso durante o tratamento, especialmente em pacientes que aparentam maior robustez do que realmente possuem^32^.

A dinâmica da síndrome da fragilidade é pautada por mecanismos de transição que devem ser criteriosamente inferidos a partir da observação clínica. Evidências sugerem que a progressão da neoplasia, a toxicidade inerente ao tratamento antineoplásico e a exacerbação de comorbidades pré-existentes ou intercorrências agudas atuam como gatilhos para o desenvolvimento ou agravamento desse estado. Embora a fragilidade possua um caráter mutável e seja passível de intervenção, as diretrizes da *International Society of Geriatric Oncology* (SIOG) e da *American Society of Clinical Oncology* (ASCO) enfatizam a necessidade de monitoramento contínuo durante e após o ciclo terapêutico^33^ ^-^ ^34^. Nesse cenário, a capacitação técnica do enfermeiro torna-se um pilar estratégico, permitindo a identificação precoce de declínios funcionais e a implementação de guias de monitoramento especializados, essenciais para a mitigação de riscos e a promoção da segurança do paciente idoso.

No contexto da prática clínica rotineira, o monitoramento longitudinal da fragilidade - pautado em reavaliações periódicas após a instauração do regime oncológico e integrado a intervenções geriátricas não oncológicas - constitui uma ferramenta indispensável para mensurar a responsividade do paciente ao plano terapêutico. Essa vigilância contínua fornece subsídios críticos para que a equipe multidisciplinar proceda ao ajuste dinâmico das condutas, permitindo a manutenção, intensificação, desescalonamento ou interrupção das intervenções conforme a evolução do quadro clínico. Tal abordagem viabiliza o redirecionamento estratégico do foco assistencial para vulnerabilidades emergentes, que podem transpor as prioridades identificadas no estágio pré-tratamento, assegurando uma assistência personalizada e centrada na trajetória clínica da pessoa idosa^35^.

No que concerne à funcionalidade, a casuística do presente estudo concentrou-se nos níveis 2 e 3 da escala ECOG, evidenciando que o comprometimento do *status* de desempenho atua como um preditor direto do incremento no risco de toxicidade na população idosa. Em contrapartida, um estudo alemão conduzido com 44 idosos, embora tenha identificado uma prevalência de 15,9% para os mesmos graus de ECOG (2 e 3), não observou associação estatisticamente significante com o risco de eventos adversos (p = 0,310)^22^. Por outro lado, outro estudo identificou uma correlação positiva entre a síndrome da fragilidade, avaliado pelo índice de fragilidade, e a ECOG em pessoas idosas com câncer, aumentando 0,10 pontos da escala da fragilidade quando o paciente estiver classificado com o ECOG 0 a 1^24^. 

A análise exploratória de segurança em ensaios clínicos baseados na escala ECOG corrobora a premissa de que o declínio funcional é um preditor de desfechos desfavoráveis. Observou-se que taxas crescentes de eventos adversos estão intrinsecamente associadas ao comprometimento do *status* de desempenho, resultando em índices elevados de descontinuação prematura do tratamento. Esse cenário de vulnerabilidade funcional não apenas limita a continuidade do protocolo antineoplásico, como também atua como um fator determinante para o agravamento do quadro clínico ou o óbito, reforçando a necessidade de monitoramento rigoroso em pacientes com menor reserva funcional. A ASCO, em uma declaração, recomendou a inclusão de pacientes com capacidade funcional reduzida para a participação em ensaios clínicos, já que isso limita as opções disponíveis para o tratamento^36^.

Com a progressão da neoplasia, a capacidade funcional tende ao declínio acentuado, impactando severamente a qualidade de vida e consolidando-se como um robusto preditor independente de sobrevida. Esse comprometimento é exacerbado pelo estado inflamatório sistêmico e pelo manejo ineficaz da sintomatologia. Biomarcadores, como a elevação da Proteína C-Reativa (PCR) e quadros de caquexia oncológica, atuam como mediadores críticos na deterioração da reserva funcional da pessoa idosa^37^. Embora o ECOG-PS seja amplamente adotado na oncologia clínica devido à sua aplicabilidade prática e agilidade assistencial, a literatura adverte que tal instrumento pode ser insuficiente para capturar as nuances da performance geriátrica e gerontológica, negligenciando domínios específicos que compõem a multidimensionalidade do envelhecimento^30^.

O estudo apresenta limitações tais como: o método, que por ser de tipo transversal, não resulta na identificação da causalidade do risco de toxicidade, além de não ser possível fazer inferência estatística, pois a amostra foi conformada por conveniência. Mesmo com essas limitações, os resultados demonstram informações importantes sobre a pessoa idosa e como o enfermeiro pode agir antes deste apresentar toxicidade. 

Esses achados são extremamente importantes para a enfermagem porque destacam fatores que podem ajudar na identificação precoce de pacientes mais vulneráveis à toxicidade por quimioterapia. Ao saber que a pessoa idosa com mais idade, com maior fragilidade e pior estado funcional têm maior risco de desenvolver efeitos adversos, a equipe de enfermagem pode monitorar esses pacientes de forma mais cuidadosa, ajustar os cuidados e intervenções de acordo com suas necessidades específicas. Tais ações contribuem para uma assistência mais segura, individualizada e eficiente para a pessoa idosa em tratamento quimioterápico, além de possibilitar intervenções precoces que podem minimizar eventuais toxicidades, melhorar a qualidade de vida do paciente e consequentemente otimizar os resultados do tratamento oncológico.

## Conclusão

Os resultados do estudo mostram que o risco de toxicidade foi de moderado e alto na amostra e que esteve associado a uma maior idade do participante, maior fragilidade e piora do ECOG.

Esses resultados destacam a necessidade de melhoria no processo de identificação de possíveis riscos para toxicidades relacionadas ao tratamento quimioterápico em pacientes idosos no primeiro dia do tratamento quimioterápico, contribuindo para um melhor plano de cuidado e, consequentemente, melhor assistência a essa população durante o tratamento. A identificação desses riscos pode ser realizada na prática assistencial da equipe de enfermagem, pois é a equipe que tem o primeiro contato, além de já fazer avaliações pré-tratamento, como a avaliação de rede venosa, para mitigar risco de extravasamento, por exemplo. O rastreamento desse risco pode ser realizado por meio da aplicação do instrumento de Hurria e a avaliação geriátrica ampla, aplicada na consulta de enfermagem, antes do início do primeiro ciclo do tratamento quimioterápico, com o objetivo de identificar as vulnerabilidades no paciente idoso. 

Diante dos resultados, é fundamental destacar a importância de um olhar diferenciado e atento para essa população, a fim de promover um melhor andamento do tratamento, reduzir as toxicidades e pausas no tratamento, podendo melhorar a qualidade de vida das pessoas idosas durante o período terapêutico. Além disso, ressalta-se a necessidade de realizar estudos com amostras maiores, que possam proporcionar uma compreensão mais aprofundada e confirmar os achados do presente estudo. Ainda, é importante investir em pesquisas com metodologias variadas, visando a desenvolver intervenções baseadas em evidências sólidas e claras, que sejam adequadas às especificidades da população idosa, com a finalidade de aprimorar o cuidado e oferecer uma prática assistencial mais segura e eficaz.

## Data Availability

Os conjuntos de dados relacionados a este artigo estarão disponíveis mediante solicitação ao autor correspondente.

# Translation: Riesgo de toxicidad en adultos mayores sometidas a quimioterapia y sus factores asociados^*^

**DOI:** 10.1590/1518-8345.7910.4987

**Authors:** Rianne Rodrigues de Lira, Alexandre de Souza Santos, Bruna Andrade Vigo, Jack Roberto Silva Fhon

**Affiliations:** 1 Universidade de São Paulo, Escola de Enfermagem, São Paulo, SP, Brasil.

**Keywords:** Anciano, Geriatría, Quimioterapia, Efectos Colaterales y Reacciones Adversas Relacionados con Medicamentos, Toxicidad, Enfermería.

## Abstract

**Objetivo:**

analizar el riesgo de toxicidad y los factores asociados en adultos mayores sometidos a quimioterapia.

**Método:**

estudio transversal y analítico que comprendió 179 participantes. La recolección de datos utilizó un formulario de perfil demográfico y revisión de las historias clínicas, complementado con instrumentos validados para la evaluación geriátrica y oncológica: *10-point Cognitive Screener,* Escala de Depresión Geriátrica*,* Puntuación de Toxicidad de Hurria, ECOG, *Simple Questionnaire to Rapidly Diagnose Sarcopenia*, Escala de Fragilidad de Tilburg y Escala de Apoyo Social. El procesamiento de datos se basó en métodos estadísticos analíticos inferenciales, adoptándose para todas las pruebas estadísticas un nivel de significancia estadística de p < 0,05.

**Resultados:**

el estudio presentó predominio de mujeres (58,4%), con un promedio de edad de 67,71 años, jubiladas/os (62,9%), presencia de cuidador (88,8%), deterioro cognitivo (31,4%), ECOG grados 2 y 3 (20,1%), riesgo de sarcopenia (33,7%), fragilidad (56,2%), síntomas depresivos (23%) y riesgo de toxicidad moderado y alto (73,6%). Se identificó que mayor edad, mayor fragilidad y peor rendimiento funcional aumentaron el riesgo de toxicidad.

**Conclusión:**

el riesgo de toxicidad asociado a la quimioterapia está presente en los adultos mayores; sumándose a la edad, el deterioro del estado funcional y la fragilidad. Este hallazgo refuerza la importancia de una mejor identificación de los diagnósticos de enfermería, lo que permite mejorar el plan de cuidados.

## Destacados

(1) Riesgo de toxicidad moderado y alto asociado al aumento de la edad.

(2) Riesgo de toxicidad moderado y alto asociado a una mayor fragilidad.

(3) Riesgo de toxicidad moderado y alto asociado a una peora en el rendimiento funcional.

(4) Escasez de estudios en adultos mayores con cáncer, de entre 60 y 70 años.

## Introducción

Con los cambios demográficos, la pirámide de población se ha modificado y el número de personas mayores de 60 años ha aumentado^1^. En 2020, el escenario mundial del cáncer registró 19,3 millones de casos incidentes, de los cuales el 64% afectó a personas de 60 años o más, un grupo que representó el 71,3% de la mortalidad asociada con estas patologías. Las neoplasias diagnosticadas más prevalentes incluyeron sitios pulmonares, colorrectales, prostáticos, mamarios y gástricos. Las proyecciones epidemiológicas indican un empeoramiento sustancial de esta situación: se estima que, para 2040, la incidencia anual alcanzará los 20,7 millones de casos nuevos, con un contingente de 12,7 millones de muertes, lo que destaca el creciente impacto de las enfermedades neoplásicas en las poblaciones de adultos mayores en todo el mundo^2^.

En el contexto epidemiológico brasileño, las proyecciones para el trienio 2023-2025 estimaron una incidencia de 483.000 nuevos casos de cáncer. El análisis de la distribución por localizaciones primarias reveló un predominio de neoplasias de mama (10,5%) y próstata (10,2%), seguidas, en términos de prevalencia, por tumores de colon y recto (6,5%)^3^. Estos datos reiteran la necesidad de políticas públicas dirigidas a la detección y el diagnóstico precoz de estas patologías, que representan la principal carga de morbilidad neoplásica en el territorio nacional.

Las patologías oncológicas, así como sus respectivos protocolos terapéuticos, desencadenan repercusiones multidimensionales que trascienden el cuadro clínico e impactan en las esferas psicosocial y socioeconómica de los pacientes, sus núcleos familiares y comunidades. Se observa que los sistemas de salud en las economías emergentes (de ingresos bajos y medios) presentan vulnerabilidades estructurales en el manejo de esta carga epidemiológica. Este escenario se agrava por la inequidad en el acceso, donde una porción sustancial de la población mundial permanece marginada en cuanto al diagnóstico temprano y las intervenciones terapéuticas calificadas dentro de los plazos oportunos, lo que compromete el pronóstico y la sostenibilidad de los servicios de salud^4^.

Las modalidades terapéuticas aplicadas a adultos y adultos mayores incluyen enfoques aislados o combinados, basados en intervenciones quirúrgicas, radioterapia y farmacoterapia especializada; esta última abarca quimioterapia citotóxica, terapia hormonal, inmunoterapia y terapias dirigidas molecularmente. Dentro del ámbito de los tratamientos sistémicos, la quimioterapia antineoplásica se emplea bajo diferentes guías clínicas como modalidad curativa; adyuvante, con el objetivo de erradicar las micrometástasis residuales después de la intervención definitiva; neoadyuvante, con el objetivo de reducir el estadio tumoral antes del procedimiento principal; o también, con capacidad paliativa, para el control de los síntomas y la extensión de la sobrevida con calidad^5^.

Durante el tratamiento, el paciente está sujeto a la manifestación de eventos adversos, conceptualizados como respuestas iatrogénicas nocivas e involuntarias, resultantes de la administración de fármacos en dosis habituales con fines profilácticos, diagnósticos o terapéuticos. Tales eventos requieren una monitorización rigurosa, puesto que la toxicidad sistémica de los agentes antineoplásicos con frecuencia trasciende el tumor, afecta tejidos sanos y exige intervenciones de apoyo para mantener la viabilidad del tratamiento^6^.

Estos efectos adversos son inherentes al mecanismo de acción inespecífico de la quimioterapia, que no posee selectividad absoluta para las células neoplásicas. La toxicidad se manifiesta predominantemente en tejidos caracterizados por una alta tasa de proliferación celular, si bien la farmacodinámica de estos agentes puede desencadenar disfunciones en diversos sistemas orgánicos. La magnitud de estas reacciones es variable, dependiendo de la especificidad del protocolo de quimioterapia, la reserva funcional del paciente y el grado de toxicidad sistémica ejercida sobre las células sanas^7^.

La medición de la toxicidad derivada de la terapia oncológica es un paso fundamental en la práctica clínica del equipo sanitario. Para ello, se adopta el *Common Terminology Criteria for Adverse Events* (CTCAE), un protocolo establecido y actualizado periódicamente por el *National Cancer Institute* (NCI). Esta herramienta proporciona una taxonomía descriptiva para clasificar los eventos adversos y los estratifica en cinco niveles de gravedad según la gravedad clínica y el impacto funcional, lo que permite la estandarización de las prácticas de manejo y la comparabilidad de los datos en la investigación clínica^8^.

En 2018, la *American Society of Clinical Oncology* (ASCO) ratificó la indispensabilidad de la Valoración Geriátrica Integral (VGI) para pacientes adultos mayores sometidos a quimioterapia. La guía sugiere que la aplicación de esta herramienta multidimensional es esencial para respaldar una planificación terapéutica personalizada, trascendiendo el análisis cronológico aislado. La implementación de la VGI permite la estratificación del riesgo y la predicción de eventos adversos potencialmente evitables, además de optimizar la seguridad del paciente y contribuir en la toma de decisiones clínicas frente a las vulnerabilidades intrínsecas al envejecimiento^9^.

Los estudios han demostrado que la prevalencia de toxicidad en adultos mayores es elevada, situándose entre el 46%^10^ y el 56,0%^11^. Asimismo, el riesgo se asocia al deterioro cognitivo^12^, a la disminución del Índice de Masa Corporal (IMC)^10^ y al síndrome de fragilidad^13^, factores que pueden conllevar riesgos para la salud.

La toxicidad quimioterápica en los adultos mayores es de suma importancia debido a sus particularidades fisiológicas, como alteraciones en la función renal, hepática e inmunológica, además de posibles comorbilidades y la polifarmacia prevalente. Estas particularidades pueden incrementar el riesgo de efectos adversos y complicaciones durante el tratamiento, por lo que es fundamental que la enfermería comprenda estos factores para brindar una atención más segura y eficaz. Al investigar esta toxicidad, la enfermería puede desarrollar estrategias de monitorización, intervención temprana y educación del paciente, además de promover una atención más personalizada, capaz de minimizar los efectos secundarios y mejorar la calidad de vida del adulto mayor durante el tratamiento oncológico. En este sentido, el objetivo de este estudio fue analizar el riesgo de toxicidad y los factores asociados en adultos mayores sometidos a quimioterapia.

## Método

### Tipo de estudio

Estudio cuantitativo, observacional, analítico y de corte transversal.

### Lugar de estudio

El estudio se realizó en el *Instituto do Câncer do Estado de São Paulo* (ICESP), una unidad del complejo *Hospital das Clínicas* de la Facultad de Medicina de la *Universidade de São Paulo* (HCFMUSP). La elección de este entorno de investigación se justifica por su reconocimiento como centro oncológico de referencia en América Latina, caracterizado por su alta complejidad asistencial y su servicio especializado a la población adulta mayor dentro del Sistema Único de Salud (SUS).

El hospital está ubicado en la región central de la capital y opera bajo un régimen de atención integral dentro del Sistema Único de Salud (SUS) y es el responsable de brindar atención a aproximadamente el 10% de la demanda oncológica del estado.

La unidad de terapia antineoplásica ambulatoria, ubicada en el undécimo piso, cuenta con una infraestructura diseñada con 107 estaciones de infusión. Esta capacidad instalada permite la implementación de diversos protocolos terapéuticos, como la administración de quimioterapia, terapia hormonal, inmunoterapia y otras intervenciones sistémicas^14^.

### Período de recopilación de datos

La recopilación de datos se llevó a cabo entre julio y octubre de 2024, en horario matutino y vespertino, de lunes a viernes, por el investigador y dos colaboradores. Todos los participantes recibieron capacitación previa, mediante reuniones virtuales y presenciales, sobre la aplicación y cumplimentación de los instrumentos, además de contar con acompañamiento y supervisión tanto al inicio de la recopilación de datos como durante el abordaje y el seguimiento de las entrevistas por el investigador principal.

### Población y muestra

La población de estudio estuvo compuesta por adultos mayores en la fase inicial de un protocolo de quimioterapia e incluyó tanto a personas sin tratamiento previo (*native*) como a aquellas que iniciaban un nuevo ciclo de tratamiento tras una recaída o progresión de la enfermedad. Todos los participantes se encontraban en el primer día del nuevo ciclo. Se adoptó un método de muestreo por conveniencia, delimitado por el período de recolección de datos.

El proceso de selección consistió en una evaluación sistemática realizada por el investigador principal, basada en el cronograma clínico de la unidad de quimioterapia, donde se verificaron criterios como la edad, el ciclo actual y el régimen terapéutico. Los pacientes elegibles se ingresaron en una base de datos compartida, lo que optimizó la planificación de las visitas presenciales y garantizó la sistematización del flujo de muestreo, de acuerdo con el cronograma de atención de la institución.

Durante la fase de recopilación de datos, se evaluaron 325 participantes potenciales. De estos, se logró contactar a 238 personas, de las cuales 22 rechazaron participar. Además, se excluyó a 17 por no cumplir con los criterios de elegibilidad y a 20 por no completar el protocolo terapéutico en la fecha prevista. Por consiguiente, la muestra final estuvo compuesta por 179 participantes.

### Criterios de selección

Para la composición de la muestra del estudio, se establecieron los siguientes criterios de elegibilidad: a) individuos de ambos sexos con una edad cronológica igual o superior a 60 años; b) diagnóstico histopatológico de neoplasia sólida primaria; c) inicio del protocolo de quimioterapia intravenosa, incluyendo tanto a los pacientes en tratamiento de primera línea como a aquellos sometidos a una alternancia del régimen terapéutico (cambio de línea) identificados en la historia clínica electrónica como C1D1, es decir, el primer día del primer ciclo de tratamiento de quimioterapia; y capacidad para comunicar y respuesta a cuestionarios. Los criterios de exclusión fueron: tener más de un diagnóstico oncológico en tratamiento concomitante; tener diagnóstico oncohematológico; y hallarse incluido en protocolos de investigación clínica.

### Variables del estudio

La variable dependiente del estudio, el riesgo de toxicidad, se analizó numéricamente y se asoció con variables demográficas, clínicas, de estado cognitivo, de rendimiento funcional, de síntomas depresivos, de fragilidad, de sarcopenia y de apoyo social.

### Recolección de datos

Las entrevistas se realizaron con los participantes una vez que se acomodaron en el ambiente donde recibirían el tratamiento de quimioterapia. Las entrevistas tuvieron lugar mientras se preparaba la medicación para su administración, durante un período de aproximadamente una hora. Cada entrevista duró un promedio de 35 a 45 minutos, lo que garantizó un tiempo suficiente para recopilar información detallada y fiable.

### Instrumentos para la recolección de la información

Se utilizaron los siguientes instrumentos:

Para la caracterización de la muestra, se utilizó un formulario estructurado para recopilar datos sociodemográficos y del perfil económico. Este instrumento permitió obtener variables fundamentales, como sexo, edad cronológica, estado civil, raza o color autodeclarado y nivel educativo. Además, se investigaron indicadores del contexto de vida y el apoyo social, como la actividad laboral actual, los ingresos familiares mensuales, la configuración de la vivienda y la presencia de apoyo de cuidadores, lo que garantizó una comprensión integral de los determinantes sociales que pueden influir en el resultado clínico de los adultos mayores.

La recolección de los datos clínicos se realizó mediante el análisis de historias clínicas, con el objetivo de identificar variables oncológicas fundamentales para el análisis del pronóstico y el riesgo terapéutico. Se extrajo información sobre la localización primaria de la neoplasia, la estadificación clínica inicial, la línea de tratamiento actual y el protocolo de quimioterapia prescripto. Además, se verificó el historial de exposición previa a la inmunoterapia y los criterios de elegibilidad para la administración de quimioterapia citotóxica, lo que garantizó un registro preciso de la trayectoria terapéutica de cada participante.

El desempeño cognitivo se evaluó por medio del *10-point Cognitive Screener* (10-CS) creado en Brasil y utilizado como medida rápida de desempeño cognitivo. Esta prueba se compone de subpruebas de orientación temporal (día, mes y año) (0 a 3 puntos), aprendizaje y evocación de tres objetos (0 a 3 puntos) y fluidez verbal categórica (animales en 60 segundos) (0 a 4 puntos).

Se suman dos puntos por personas sin escolarización y un punto por personas con entre uno y tres años de escolarización. La puntuación total varía de cero a 10, y se clasifica al participante como: normal (10 - 8 puntos), posible deterioro cognitivo (6 - 7 puntos) y probable deterioro cognitivo (0 - 5 puntos)^15^.

Los síntomas depresivos se evaluaron utilizando la Escala de Depresión Geriátrica (EDG), que ha sido validada para el portugués brasileño^16^. La escala consta de 15 preguntas dicotómicas (Sí y No); la puntuación varia de 0 a 15 y presenta el punto corte ≥ 5 como indicación de casos de probable presencia de síntomas depresivos.

El riesgo de toxicidad se identificó utilizando la Puntuación de Toxicidad Hurria, traducida y validada en Brasil^17^. Este es un instrumento predictivo para evaluar el riesgo de toxicidad, que abarca 11 variables, a saber: edad mayor o igual a 72 años; cáncer del tracto gastrointestinal o genitourinario; número de agentes quimioterapéuticos; niveles de hemoglobina inferiores a 11 g/dL en hombres y 10 g/dL en mujeres; aclaramiento de creatinina menor a 34 mL/min; audición limítrofe o pérdida auditiva severa; haber sufrido una o más caídas en los últimos seis meses, requerir asistencia parcial para la toma de medicamentos o no poder hacerlo, limitación moderada o marcada para caminar una cuadra; y la disminución de las actividades sociales, al menos en ocasiones, debido a limitaciones físicas o emocionales^17^.

El aclaramiento de creatinina se determinó a partir de la fórmula de Cockcroft-Gault, mediante calculadora electrónica proporcionada por la Sociedad Brasileña de Nefrología (SBN). Para el cálculo, se consideraron el peso corporal, la edad cronológica y el valor de creatinina sérica obtenido al momento de la autorización del protocolo de quimioterapia. De manera similar, los niveles de hemoglobina se extrajeron de las pruebas de laboratorio vinculadas a la autorización de la terapia citotóxica. La estratificación del riesgo de toxicidad se estableció a partir de la suma de las puntuaciones de las variables predictoras, lo que permitió clasificar a los participantes en tres categorías: riesgo bajo (0-5 puntos), riesgo moderado (6-9 puntos) y riesgo alto (10-19 puntos)^17^.

El estado funcional se evaluó utilizando la escala de la *Eastern Cooperative Oncology* (ECOG-PS). Este instrumento, ampliamente validado en oncología clínica, mide el grado de autonomía del paciente en cinco niveles: grado 0 (totalmente activo); grado 1 (restricciones para realizar actividades intensas, pero capaz de realizar trabajo ligero); grado 2 (capaz de realizar el autocuidado, pero incapaz de realizar actividades laborales, permaneciendo fuera de la cama durante más del 50% del tiempo de vigilia); grado 3 (autocuidado limitado, permaneciendo en la cama o en una silla durante más del 50% del tiempo de vigilia); y grado 4 (incapacidad total para el autocuidado y confinamiento completo a la cama)^18^.

La sarcopenia se identificó por el *Simple Questionnaire to Rapidly Diagnose Sarcopenia* (SARC-F), validado para Brasil y compuesto por cinco preguntas objetivas sobre fuerza, deambulación y acción de levantarse de una silla, subir un tramo de escaleras y haber sufrido caídas; además de la verificación de la circunferencia de la pantorrilla, cuyo valor puede oscilar de 0 a 20 puntos. Como resultado, el participante puede ser clasificado como sin signos sugestivos de sarcopenia (0 a 10 puntos) y con signos sugestivos de sarcopenia (11 a 20 puntos)^19^.

La evaluación de la fragilidad se realizó a través de la Escala de Fragilidad de Tilburg (sigla en inglés TFI), un instrumento validado y adaptado transculturalmente al idioma portugués. El TFI se caracteriza por ser una escala multidimensional compuesta por 15 ítems objetivos y autoinformados, estructurado para investigar tres dominios fundamentales: físico, psicológico y social. La puntuación total se obtiene sumando las respuestas, con un rango de 0 a 15 puntos. Se adoptó un punto de corte mayor o igual a cinco puntos para la clasificación de fragilidad, como se recomienda en la literatura, para la identificación de individuos con mayor vulnerabilidad clínica^20^. 

La percepción de apoyo social se midió por medio del *Medical Outcomes Study Social Support Survey* (MOS-SSS), en su versión adaptada y validada para la población brasileña, que consta de 19 ítems que evalúan la frecuencia de disponibilidad de apoyo a través de la pregunta central: “Si lo necesita, ¿con qué frecuencia puede contar con alguien?”. Las respuestas se estructuran en una escala Likert de cinco puntos, desde 0 (“nunca”) a 4 (“siempre”). Dada la ausencia de valores normativos específicos para el contexto brasileño, el análisis se basó en la premisa de proporcionalidad, en la que puntuaciones totales más altas indican una percepción superior de apoyo social en los dominios investigados^21^.

### Análisis de datos

El procesamiento y el análisis estadístico de los datos se llevaron a cabo con el auxilio del *software Statistical Package for the Social Sciences* (SPSS), versión 25.0. La normalidad de la distribución de las variables numéricas se verificó mediante la prueba de Kolmogorov-Smirnov. Para la caracterización de la muestra, se realizó un análisis descriptivo, expresando las variables categóricas mediante frecuencias absolutas y relativas. Las variables continuas se analizaron mediante medidas de tendencia central y dispersión, específicamente la media y la desviación estándar.

A continuación, se realizaron análisis bivariados para investigar las asociaciones con el resultado “riesgo de toxicidad”, tratado como variable dependiente. Se aplicó la prueba de Chi-cuadrado de Pearson para tabular las variables categóricas. Se utilizó la prueba t de Student para muestras independientes para comparar las medias entre distintos grupos, con el fin de identificar la significación estadística en las correlaciones establecidas entre los factores predictores y la vulnerabilidad al evento adverso.

Para determinar la magnitud de las asociaciones entre el riesgo de toxicidad (variable dependiente) y los factores predictivos, se aplicó un modelo de regresión lineal múltiple. Las variables independientes incluyeron el perfil demográfico (edad, sexo, estado civil, presencia de un cuidador, nivel educativo, paridad, ingresos y apoyo social), indicadores de salud (comorbilidades) y dominios clínicos (capacidad funcional, estado cognitivo, sintomas depresiva, sarcopenia y fragilidad). En todos los análisis inferenciales, se adoptó un nivel de significancia de p < 0,05 y un intervalo de confianza (IC) del 95%.

### Aspectos éticos

El protocolo de investigación fue presentado y aprobado por el Comité Científico de Docencia e Investigación del ICESP (registro NP5049/23) y por el Comité de Ética en Investigación del Hospital das Clínicas de la Facultad de Medicina de la *Universidade de São Paulo* (CEP-HCFMUSP), bajo el Certificado de Presentación de Evaluación Ética (CAAE) nº 78467124.2.0000.0068.

El estudio se realizó en estricto cumplimiento de los preceptos éticos establecidos en la Resolución n.° 466/2012 del Consejo Nacional de Salud, que rige la investigación con seres humanos. La recolección de datos comenzó únicamente después de que todos los participantes aceptaran y firmaran el Consentimiento Informado (CI), lo que garantiza el anonimato y el carácter voluntario de la colaboración.

### Resultados

La población estudiada mostró un predominio de mujeres (58,4%), de entre 60 y 69 años (63,5%), con un promedio de edad de 67,7 años (= 5,63), casadas (47,2%), jubiladas (62,9%), con un ingreso mensual promedio de R$ 2.709,84 (= 2.426,50) y 8,66 (= 4,54) años de escolaridad, con vivienda propia (71,9%) y con un cuidador (88,8%) (Tabla 1).

El número promedio de comorbilidades fue de 1,26 (DE=1,09), siendo las más prevalentes la hipertensión arterial sistémica (50,6%), la diabetes mellitus (28,1%) y la dislipidemia (21,3%), seguidas de cardiopatía e hipotiroidismo (ambos con un 3,4%).

Según los registros de consultas médicas, el diagnóstico de cáncer más común entre los participantes fue el de mama (18,5%), seguido del de bronquios y pulmones (12,9%), colon (11,8%), recto (7,3%), páncreas (6,2%), estómago (3,9%), orofaringe y esófago (3,4%, respectivamente).

**Tabla 1:** Características sociodemográficas de los adultos mayores el primer día de tratamiento de quimioterapia (n = 179). São Paulo, SP, Brasil, 2024

Variable	Categoría	n* (%)	Media (=DE^†^)
Sexo	Femenino	104 (58,4)	
	Masculino	74 (41,6)	
Edad	60 - 69	113 (63,5)	67,71 (= 5,63)
	70 - 79	58 (32,6)	
	80 o más	7 (3,9)	
Estado civil	Casado	84 (47,2)	
	Soltero	34 (19,1)	
	Viudo	33 (18,5)	
	Divorciado	21 (11,8)	
	Separado	5 (2,8)	
	Relación estable	1 (0,6)	
Etnia o raza autodeclarada	Blanca	96 (53,9)	
	Mestiza	46 (25,8)	
	Negra	32 (18,0)	
	Indígena	2 (1,1)	
	Amarilla	2 (1,1)	
Actividad actual	Jubilado	112 (62,9)	
	Desempleado	35 (19,7)	
	Autónomo	19 (10,7)	
	Trabaja	12 (6,7)	
Educación			8,66 (= 4,54)
Ingresos mensuales			2.709,84^‡^ (= 2.426,55)
Presencia de cuidador	Sí	158 (88,8)	
	No	20 (11,2)	
Tipo de vivienda	Vivienda propia	128 (71,9)	
	Vivienda alquilada	27 (15,2)	
	Vivienda del cuidador principal	20 (11,2)	
	Otros	3 (1,7)	

*n = Número; ^†^DE = Desviación estándar; ^‡^Salario mínimo actual = R$ 1.412,00, Brasil, 2024

En cuanto a la caracterización de las modalidades terapéuticas, la mayoría de la muestra (82,2%) no tenía antecedentes de exposición a la inmunoterapia, mientras que el 11,8% sí la había recibido. Respecto a los protocolos de quimioterapia, predominó la quimioterapia de primera línea (33,7%), seguida de los regímenes adyuvantes (18,5%) y neoadyuvantes (11,3%). Las líneas de tratamiento posteriores se identificaron en menor proporción: segunda línea (9,6%), tercera línea (6,7%) y cuarta línea (2,2%). Además, las intenciones terapéuticas y los protocolos específicos registrados fueron tratamiento paliativo (9,0%), terapia de inducción (5,1%), protocolos estándar (3,9%) e intención curativa (1,7%).

En cuanto a la evaluación clínica, se evidenció que los participantes presentaban posible deterioro cognitivo (20,8%) y probable deterioro cognitivo (9,6%), con categoría ECOG 2 (19,1%), riesgo de sarcopenia (33,7%), fragilidad (56,2%), riesgo de toxicidad moderado (48,9%) y alto (24,7%), y con síntomas depresivos (23,0%). En cuanto al apoyo social, se encontró en el dominio apoyo material con alta percepción (84,3%), apoyo afectivo con alta percepción (89,3%), apoyo emocional con alta percepción (75,8%) e interacción social positiva con alta percepción (69,7%) (Tabla 2).

**Tabla 2:** Caracterización de la evaluación de los adultos mayores el primer día de tratamiento de quimioterapia (n = 179). São Paulo, SP, Brasil, 2024

Variable	Categoría	Media (=DE*)	n^†^	%
Estado cognitivo	Normal	7,89 (1,86)	124	69,7
	Posible deterioro cognitivo		37	20,8
	Probable deterioro cognitivo		17	9,6
ECOG-PS^‡^	0	0,93 (0,72)	51	28,7
	1		91	51,1
	2		34	19,1
	3		2	1,1
Sarcopenia	Sin riesgo de sarcopenia	6,92 (5,69)	118	66,3
	Con riesgo de sarcopenia		60	33,7
Fragilidad	No frágil	5.11 (2.36)	79	43,8
	Frágil		100	56,2
Hurria	Bajo	7,57 (3,03)	47	26,4
	Moderado		87	48,9
	Alto		44	24,7
Síntomas depresivos	Sin síntomas	2,96 (2,59)	137	77,0
	Con síntomas		41	23,0
MOSS-SSS^§^	Apoyo material	14,67 (3,00)		
	Alta percepción		150	84,3
	Percepción promedio		22	12,4
	Baja percepción		6	3,4
	Apoyo emocional	11,39 (1,93)		
	Alta percepción		159	89,3
	Percepción promedio		14	7,9
	Baja percepción		5	2,8
	Apoyo emocional	26,88 (9,71)		
	Alta percepción		135	75,8
	Percepción promedio		20	11,2
	Baja percepción		23	12,9
	Interacción social positiva	12,65 (5,37)		
	Alta percepción		124	69,7
	Percepción promedio		21	11,8
	Baja percepción		33	18,5

*DE = Desviación estándar; ^†^n = Número; ^‡^ECOG-PS = *Eastern Cooperative Oncologic Group Performance Status Scale*; ^§^MOSS-SSS = Escala de Apoyo Social

Al investigar las relaciones entre los resultados de toxicidad y las variables independientes categóricas, la aplicación de la prueba t de *Student* reveló una disparidad estadísticamente significativa en el riesgo de desarrollar eventos adversos de quimioterapia en función del sexo (p = 0,04) (Tabla 3).

En cuanto a la prueba de correlación, se demostró que el riesgo de toxicidad estaba correlacionado con la edad (p < 0,001), ECOG (p < 0,001), síntomas depresivos (p < 0,001), sarcopenia (p < 0,001) y fragilidad (p < 0,001) (Tabla 4).

Los análisis de asociación revelaron que el riesgo de toxicidad por quimioterapia estaba significativamente influenciado por variables biológicas y funcionales. Se observó que la edad avanzada (p = 0,001), el aumento del escore de fragilidad (p = 0,012) y el deterioro del estado funcional, medido por la escala ECOG (p = 0,003), fueron predictores positivos de una mayor vulnerabilidad a los eventos adversos durante el tratamiento (Tabla 5).

**Tabla 3:** Relación entre el riesgo de toxicidad relacionada con la quimioterapia y el sexo, el estado civil y la presencia de cuidador en los adultos mayores el primer día de tratamiento de quimioterapia (n = 179). São Paulo, SP, Brasil, 2024

Variable	Categoría	Media	=DE*	valor p^†^
Sexo	Femenino	7,16	2,78	0,04
	Masculino	8,14	3,03	
Estado civil	En pareja	7,99	3,14	0,07
	Sin pareja	7,18	2,89	
Presencia de cuidador	Sí	7,71	2,95	0,14
	No	6,45	3,53	

*DE = Desviación estándar; ^†^valor p = Significancia estadística

**Tabla 4:** Correlación entre el riesgo de toxicidad relacionada con la quimioterapia y variables sociodemográficas y clínicas en los adultos mayores el primer día de tratamiento quimioterápico (n = 179). São Paulo, SP, Brasil, 2024

Variable	r*	valor p^†^
Edad	0,339	<0,001
Educación	-0,092	0,220
Cantidad de hijos	0,081	0,283
Ingresos de los adultos mayores	-0,039	0,602
Número total de comorbilidades	0,078	0,301
ECOG-PS^‡^	0,429	<0,001
Estado cognitivo - Escore total	-0,119	0,113
Síntomas depresivos - Escore total	0,291	<0,001
Sarcopenia - Escore total	0,332	<0,001
Fragilidad - Escore total	0,336	<0,001
MOSS-SSS^§^ Apoyo material	0,045	0,549
MOSS-SSS^§^ Apoyo afectivo	-0,143	0,058
MOSS-SSS^§^ Apoyo emocional	-0,088	0,243
MOSS-SSS^§^ Interacción social positiva	-0,126	0,093

*r = Correlación de Pearson; ^†^Valor p = Significancia estadística; ^‡^ECOG-PS = *Eastern Cooperative Oncologic Group Performance Status Scale*; ^§^MOSS-SSS = Escala de Apoyo Social

**Tabla 5:** Asociación entre el riesgo de toxicidad relacionada con la quimioterapia y variables sociodemográficas y clínicas en los adultos mayores el primer día de tratamiento quimioterápico (n = 179). São Paulo, SP, Brasil, 2024

Variable	B*	t^†^	valor p^‡^	IC 95% ^§^inf^||^ y sup^¶^	
Edad	0,128	3,379	0,001	0,053	0,202
Sexo	-0,622	-1,434	0,154	-1,479	0,235
Estado civil	-0,486	-1,171	0,244	-1,305	0,334
Presencia de cuidador	1,054	1,549	0,123	0,290	2,397
Educación	0,043	0,846	0,399	-0,057	0,143
Cantidad de hijos	0,028	0,235	0,815	-0,208	0,265
Ingresos	-5,793	-0,655	0,513	0,000	0,000
Número total de comorbilidades	0,048	0,243	0,808	-0,340	0,436
ECOG-PS**	0,957	2,983	0,003	0,323	1,590
Estado cognitivo - Escore total	0,065	0,548	0,585	-0,171	0,301
Síntomas depresivos - Escore total	0,124	1,410	0,161	-0,050	0,297
Sarcopenia - Escore total	0,064	1,644	0,102	-0,013	0,141
Fragilidad- Escore total	0,238	2,550	0,012	0,054	0,422
MOS-SSS^†† -^ Apoyo material	0,077	0,999	0,319	-0,075	0,229
MOS-SSS^††^ - Apoyo afectivo	-0,055	-0,485	0,628	-0,279	0,169
MOS-SSS^††^ - Apoyo emocional	-0,025	-1,080	0,282	-0,070	0,020
MOS-SSS^††^ - Interacción social positiva	-0,040	-0,988	0,325	-0,121	0,040

*B = Beta; ^†^t = valor t; ^‡^valor p = Significancia estadística; ^§^IC del 95 % = Intervalo de confianza; ^||^inf = Límite inferior; ^¶^sup = Límite superior; **ECOG-PS = *Eastern Cooperative Oncologic Group Performance Status Scale*; ^††^ MOSS-SSS = Escala de Apoyo Social

## Discusión

Este estudio destaca información importante relacionada con la población de adultos mayores sometidas a quimioterapia, lo que puede contribuir a una mejor gestión y planificación de la atención para estos pacientes. El estudio identificó que los adultos mayores con menor capacidad funcional y fragilidad presentan un mayor riesgo de toxicidad durante el tratamiento con quimioterapia.

En cuanto a la población estudiada, estaba compuesta principalmente por mujeres con una edad promedio de 67,7 años, con predominio del rango de edad de 60 a 69 años, y de personas casadas. Estudios similares han identificado resultados divergentes con respecto al sexo^22^ ^-^ ^23^. En cuanto a la edad, los promedios fueron similares^22^ ^-^ ^23^; lo mismo se identificó para las personas casadas^21^ ^,^ ^24^_._

En lo que respecta a la prevalencia del riesgo de toxicidad, casi tres cuartas partes de la muestra presentaba riesgo moderado o alto. Un estudio chino identificó, en 253 pacientes adultos mayores con cáncer, que el 24,17% presentó toxicidad^25^. Por otro lado, una revisión sistemática identificó, para todos los tipos de cánceres no hematológicos, una prevalencia de toxicidad del 45,7% en la población estudiada^26^. Al interpretar el valor clínico de estos datos, debe considerarse que, en la mayoría de los casos, la toxicidad no hematológica tiene un impacto en la calidad de vida del paciente, al interferir en la capacidad funcional, la esfera emocional, la función cognitiva y el uso de recursos de salud y hospitalización, lo que conlleva un mayor riesgo de institucionalización^27^.

Los estudios han identificado que el uso de herramientas específicas en la evaluación del adulto mayor, como la VGI, reduce el riesgo de toxicidad de la quimioterapia y mejora la calidad de vida^28^.

En el ámbito específico de la quimioterapia, la VGI actúa como una herramienta de estratificación clínica, al permitir la categorización del perfil del adulto mayor en tres fenotipos distintos: (a) pacientes robustos, cuya reserva funcional posibilita la administración de protocolos convencionales análogos a los de grupos de edad más jóvenes; (b) pacientes frágiles, en quienes la acumulación de comorbilidades y el deterioro funcional sugieren que el beneficio de la quimioterapia citotóxica se ve superado por los riesgos, priorizando los enfoques paliativos; y (c) pacientes vulnerables, que presentan deficiencias en dominios específicos de la VGI, requiriendo intervenciones gerontológicas personalizadas y medidas profilácticas para optimizar su viabilidad terapéutica^27^. Se enfatiza que la aplicación sistemática de esta herramienta puede ser llevada a cabo de manera eficaz por profesionales de enfermería, lo que fortalece la práctica avanzada y la gestión de riesgos en oncología.

Los análisis de asociación en este estudio confirman la edad cronológica como factor determinante, mostrando que el avance de la edad es directamente proporcional a la vulnerabilidad a eventos adversos. Sin embargo, la literatura presenta controversias; un estudio de cohorte retrospectivo realizado en Corea observó datos divergentes al estratificar la muestra en grupos de edad (menores y mayores de 70 años), sin identificar una asociación significativa entre la edad, considerada en términos aislados y el riesgo de toxicidad^23^. Esta disparidad puede atribuirse a la heterogeneidad biológica del envejecimiento, ya que los adultos mayores presentan mayores riesgos de deterioro funcional y multimorbilidad. Estas condiciones, más que la edad cronológica *per se*, resultan en una menor reserva fisiológica y una mayor interferencia en las actividades de la vida diaria y elevan la complejidad del manejo terapéutico^29^.

El manejo oncológico en la senescencia y la senilidad sigue siendo un desafío terapéutico multifactorial, condicionado por la marcada heterogeneidad clínica de este grupo de edad. Esta complejidad proviene de la variabilidad en los niveles de independencia, la carga de comorbilidades y la reserva funcional, elementos que dificultan la estandarización de las decisiones terapéuticas. Además, la senescencia fisiológica impone cambios farmacocinéticos y farmacodinámicos significativos, reduciendo la selectividad tisular y estrechando el índice terapéutico de los fármacos, lo que potencia el riesgo de toxicidad grave. La prevalencia de multimorbilidades y de la polifarmacia exacerban esta vulnerabilidad, aumentando la probabilidad de interacciones farmacológicas perjudiciales. Por último, la escasez de ensayos clínicos sólidos centrados específicamente en la población adulta mayor resulta en una brecha de evidencia con respecto a la eficacia y seguridad de los protocolos antineoplásicos, lo que limita la base para el manejo optimizado de las toxicidades^30^.

La mayor parte de la población estudiada fue categorizada como frágil y asociada con el riesgo de toxicidad. Un estudio identificó que el 43,0% de los participantes fueron clasificados como frágiles^24^. Por otro lado, otro estudio identificó que los adultos mayores consideradas frágiles no estaban asociadas con el riesgo de toxicidad^22^. La fragilidad se conceptualiza como un síndrome clínico multidimensional, caracterizado por una marcada reducción en la reserva fisiológica y la capacidad de resistir los factores estresantes. Esta condición resulta de un declive acumulativo en los sistemas fisiológicos, que se manifiesta a través de una disminución de la fuerza muscular y la funcionalidad orgánica. Como consecuencia, se observa un mayor estado de vulnerabilidad, que predispone al individuo a resultados adversos, como una disminución de la autonomía (dependencia), una mayor carga de morbilidad y mayores tasas de mortalidad^31^.

La identificación de pacientes prefrágiles y frágiles es crucial para facilitar intervenciones y evaluaciones de cuidados de apoyo más específicas y eficaces. Esta práctica tiene importantes implicaciones clínicas porque, si bien es posible reconocer a los pacientes frágiles, los cambios en los pacientes prefrágiles tienden a ser más sutiles y difíciles de detectar. Como resultado, existe el riesgo de perder oportunidades para una orientación personalizada temprana y un seguimiento más riguroso durante el tratamiento, especialmente en pacientes que parecen más robustos de lo que realmente son^32^.

La dinámica del síndrome de la fragilidad está regida por mecanismos transicionales que deben inferirse cuidadosamente a partir de la observación clínica. La evidencia sugiere que la progresión de la neoplasia, la toxicidad inherente al tratamiento antineoplásico y la exacerbación de comorbilidades preexistentes o eventos agudos actúan como desencadenantes para el desarrollo o empeoramiento de esta condición. Aunque la fragilidad es mutable y susceptible de intervención, las directrices de la *International Society of Geriatric Oncology* (SIOG) y la *American Society of Clinical Oncology* (ASCO) destacan la necesidad de un seguimiento continuo durante y después del ciclo terapéutico^33^ ^-^ ^34^. En este contexto, la formación técnica del personal de enfermería se convierte en un pilar estratégico, puesto que permite la identificación temprana de deterioros funcionales y la implementación de guías de seguimiento especializadas, esenciales para mitigar riesgos y promover la seguridad de los pacientes de edad avanzada.

En el contexto de la práctica clínica rutinaria, el seguimiento longitudinal de fragilidad -basado en reevaluaciones periódicas tras el inicio del régimen oncológico e integrado con intervenciones geriátricas no oncológicas- constituye una herramienta indispensable para medir la respuesta del paciente al plan terapéutico. Este seguimiento continuo proporciona un apoyo fundamental al equipo multidisciplinario para ajustar dinámicamente la conducta, permitiendo el mantenimiento, la intensificación, la desescalada o la interrupción de las intervenciones según la evolución del cuadro clínico. Este enfoque permite la reorientación estratégica del modelo asistencial hacia las vulnerabilidades emergentes, que pueden trascender las prioridades identificadas en la fase previa del tratamiento, asegurando una atención personalizada centrada en la trayectoria clínica del adulto mayor^35^.

En cuanto a la funcionalidad, la serie de casos del presente estudio se centró en los niveles 2 y 3 de la escala ECOG, mostrando que el deterioro del estado funcional actúa como un predictor directo de un mayor riesgo de toxicidad en la población de adultos mayores. En contrapartida, un estudio alemán realizado con 44 individuos adultos mayores, aunque identificó una prevalencia del 15,9% para los mismos niveles ECOG (2 y 3), no observó una asociación estadísticamente significativa con el riesgo de eventos adversos (p = 0,310)^22^. Por otro lado, otro estudio identificó una correlación positiva entre el síndrome de fragilidad, evaluado por el índice de fragilidad, y el ECOG en adultos mayores con cáncer, aumentando la escala de fragilidad en 0,10 puntos cuando el paciente se clasifica con ECOG 0 a 1^24^.

El análisis exploratorio de seguridad en ensayos clínicos basados en la escala ECOG corrobora la premisa de que el deterioro funcional es un predictor de resultados desfavorables. Se observó que las tasas crecientes de eventos adversos estaban intrínsecamente asociadas con el deterioro del estado funcional, lo que resultó en altas tasas de interrupción prematura del tratamiento. Este escenario de vulnerabilidad funcional no solo limita la continuidad del protocolo antineoplásico, sino que también actúa como un factor determinante para el empeoramiento del cuadro clínico o la muerte, lo que refuerza la necesidad de una monitorización rigurosa en pacientes con menor reserva funcional. En una declaración, la ASCO recomendó la inclusión de pacientes con capacidad funcional reducida para la participación en ensayos clínicos, ya que esto limita las opciones de tratamiento disponibles^36^.

Con la progresión de la neoplasia, la capacidad funcional tiende a disminuir drásticamente, impactando gravemente en la calidad de vida y estableciéndose como un predictor independiente robusto de sobrevida. Este deterioro se ve exacerbado por el estado inflamatorio sistémico y el manejo ineficaz de los síntomas. Los biomarcadores, como la proteína C reactiva (PCR) elevada y la caquexia neoplásica, actúan como mediadores críticos en el deterioro de la reserva funcional del adulto mayor^37^. Aunque el ECOG-PS es ampliamente adoptado en oncología clínica debido a su aplicabilidad práctica y agilidad en la atención, la literatura advierte que este instrumento puede ser insuficiente para capturar los matices del desempeño geriátrico y gerontológico, descuidando dominios específicos que conforman la multidimensionalidad del envejecimiento^30^.

El presente estudio presenta limitaciones, como el método. Por tratarse de un estudio transversal, no permite identificar la causa del riesgo de toxicidad. Además, no es posible realizar la inferencia estadística puesto que la selección de la muestra se dio por conveniencia. Sin embargo, a pesar de estas limitaciones, los resultados exhiben información importante sobre los adultos mayores y plantean cómo puede actuar el personal de enfermería antes de que se produzca la toxicidad.

Estos hallazgos son de gran relevancia para la enfermería, ya que resaltan factores que contribuyen a la identificación precoz de los pacientes más vulnerables a la toxicidad quimioterápica. Al saber que los adultos mayores, más frágiles y con menor capacidad funcional presentan un mayor riesgo de desarrollar efectos adversos, el equipo de enfermería puede monitorear a estos pacientes con mayor atención, ajustando los cuidados y las intervenciones según sus necesidades específicas. Tales acciones contribuyen a prestar una asistencia más segura, individualizada y eficaz a los pacientes adultos mayores sometidos a quimioterapia, además de permitir intervenciones tempranas que puedan minimizar las posibles toxicidades, mejorar la calidad de vida del paciente y, en consecuencia, optimizar los resultados del tratamiento oncológico.

## Conclusión

Los resultados del estudio muestran que el riesgo de toxicidad fue de moderado a alto en la muestra y estuvo asociado con una mayor edad de los participantes, una mayor fragilidad y un empeoramiento del estado funcional según la escala ECOG.

Estos resultados resaltan la necesidad de mejorar el proceso de identificación de riesgos potenciales de toxicidad relacionada con la quimioterapia en pacientes adultos mayores en el primer día de tratamiento, lo que contribuye a un mejor plan de cuidados y, por consiguiente, a una mejor asistencia a esta población durante el tratamiento. La identificación de estos riesgos puede llevarse a cabo en la práctica clínica del equipo de enfermería, ya que son los que tienen el primer contacto y realiza evaluaciones previas al tratamiento, como la evaluación de la red venosa, para mitigar el riesgo de extravasación, por ejemplo. La detección de este riesgo puede realizarse mediante la aplicación del instrumento Hurria y una valoración geriátrica integral, realizada durante la consulta de enfermería previa al inicio del primer ciclo de quimioterapia, con el objetivo de identificar vulnerabilidades en los pacientes adultos mayores.

Dados los resultados, es crucial destacar la importancia de un enfoque diferenciado y atento hacia esta población, con el fin de promover un mejor progreso del tratamiento, reducir la toxicidad y las interrupciones del mismo, y mejorar la calidad de vida de los adultos mayores durante el periodo terapéutico. Además, se subraya la necesidad de realizar estudios con muestras más amplias, ya que estos pueden proporcionar una comprensión más profunda y confirmar los hallazgos de este estudio. Asimismo, es importante invertir en investigación con metodologías variadas, con el objetivo de desarrollar intervenciones basadas en evidencia sólida y clara, adecuadas a las necesidades específicas de la población adulta mayor, para mejorar la atención y ofrecer una práctica asistencial más segura y eficaz.

## Data Availability

Los conjuntos de datos relacionados con este artículo estarán disponibles previa solicitud al autor correspondiente.
